# Preparation and Biochemical Activity of Copper-Coated Cellulose Nonwoven Fabric via Magnetron Sputtering and Alginate-Calcium Ion Complexation

**DOI:** 10.3390/md22100436

**Published:** 2024-09-26

**Authors:** Małgorzata Świerczyńska, Zdzisława Mrozińska, Michał Juszczak, Katarzyna Woźniak, Marcin H. Kudzin

**Affiliations:** 1 Łukasiewicz Research Network—Łódź Institute of Technology, Marii Sklodowskiej-Curie 19/27, 90-570 Lodz, Poland; 2 Institute of Polymer and Dye Technology, Faculty of Chemistry, Lodz University of Technology, Stefanowskiego 16, 90-537 Lodz, Poland; 3 Department of Molecular Genetics, Faculty of Biology and Environmental Protection, University of Lodz, Pomorska 141/143, 90-236 Lodz, Poland

**Keywords:** composite materials, cellulose, copper, alginic acid, calcium, antimicrobial properties, blood coagulation, activated partial thromboplastin time (aPTT), prothrombin time (PT), cell viability, DNA damage, PBM cells, plasmid relaxation assay

## Abstract

Alginate-based materials have gained significant recognition in the medical industry due to their favorable biochemical properties. As a continuation of our previous studies, we have introduced a new composite consisting of cellulose nonwoven fabric charged with a metallic copper core (CNW-Cu^0^) covered with a calcium alginate (ALG^−^Ca^2+^) layer. The preparation process for these materials involved three main steps: coating the cellulose nonwoven fabric with copper via magnetron sputtering (CNW → CNW-Cu^0^), subsequent deposition with sodium alginate (CNW-Cu^0^ → CNW-Cu^0^/ALG^−^Na^+^), followed by cross-linking the alginate chains with calcium ions (CNW-Cu^0^/ALG^−^Na^+^ → CNW-Cu^0^/ALG^−^Ca^2+^). The primary objective of the work was to supply these composites with such biological attributes as antibacterial and hemostatic activity. Namely, equipping the antibacterial materials (copper action on representative Gram-positive and Gram-negative bacteria and fungal strains) with induction of blood plasma clotting processes (activated partial thromboplastin time (aPTT) and prothrombin time (PT)). We determined the effect of CNW-Cu^0^/ALG^−^Ca^2+^ materials on the viability of Peripheral blood mononuclear (PBM) cells. Moreover, we studied the interactions of CNW-Cu^0^/ALG^−^Ca^2+^ materials with DNA using the relaxation plasmid assay. However, results showed CNW-Cu^0^/ALG^−^Ca^2+^’s cytotoxic properties against PBM cells in a time-dependent manner. Furthermore, the CNW-Cu^0^/ALG^−^Ca^2+^ composite exhibited the potential to interact directly with DNA. The results demonstrated that the CNW-Cu^0^/ALG^−^Ca^2+^ composites synthesized show promising potential for wound dressing applications.

## 1. Introduction

**Figure 1:**
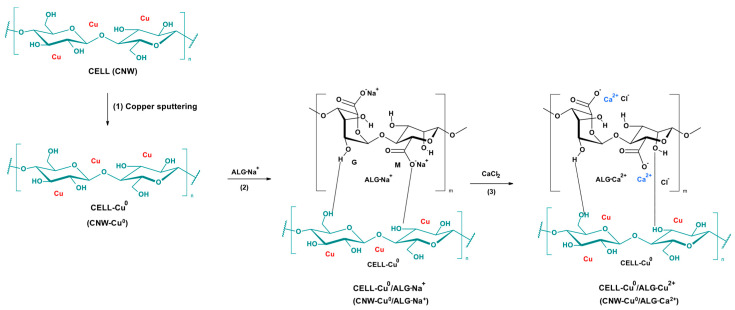
The transformation of cellulose (CNW) into the final CNW-Cu^0^/ALG^−^Ca^2+^: (1) cellulose copper sputtering (CNW → CNW-Cu^0^) (according to the Ref. [51]); (2) cellulose core covering with sodium alginate (CNW-Cu^0^ → CNW-Cu^0^/ALG^−^Na^+^); (3) subsequent crosslinking alginate upper layer with calcium ions (CNW-Cu^0^/ALG^−^Na^+^ → CNW-Cu^0^/ALG^−^Ca^2+^).

The primary challenge facing contemporary biomedical engineers is the development of a hemostatic material that not only promotes rapid blood coagulation but also demonstrates compatibility with biological tissues and possesses antibacterial properties [1]. Alginate-based biomaterials exhibit significant potential in medicine due to their advantageous properties [2,3,4]. Their ability to effectively halt bleeding makes them highly useful in situations requiring rapid intervention. Additionally, alginate materials support the wound healing process, which is crucial for the treatment of injuries and surgical procedures. Their biocompatibility ensures that they are well-tolerated by the body, minimizing the risk of allergic or rejection reactions [5,6,7,8]. Moreover, the biodegradability of these biomaterials means they decompose within the body after fulfilling their function, reducing the need for surgical removal and lowering the risk of long-term complications [9,10,11,12]. Alginate plays a critical role as a natural polymer in the development of novel medical materials. Its unique properties make it indispensable for creating advanced therapeutic solutions, with extensive applications in tissue repair and regeneration [13,14]. Alginate-based materials are gaining prominence due to their high biocompatibility and ability to facilitate rapid wound healing. These materials demonstrate excellent cell viability, enhancing their safety profile [11]. Additionally, their superior absorbency allows for effective hemostasis and the efficient absorption of substantial volumes of bodily fluids, which is crucial for the treatment of wounds with varying depths [6,15,16,17,18]. Despite its numerous advantages as a medical material, alginate lacks inherent hemostatic properties. On its own, it does not halt bleeding effectively, which is why it is frequently used in conjunction with other substances that provide the necessary hemostatic effects [19,20]. However, when sodium alginate undergoes cross-linking with divalent cations (such as Ca^2+^, Cu^2+^, Zn^2+^, or Ag^2+^), insoluble in water metal alginates are formed (e.g., ALG^−^Na^+^ + Ca^2+^→ ALG^−^Ca^2+^ + Na^+^) (Figure 1) [18,21,22,23,24,25]. These alginates, such as calcium alginate, possess procoagulant properties [26,27,28,29,30,31,32,33]. Upon contact with blood, these materials activate platelets and influence thrombin, thereby initiating the coagulation cascade. This facilitates effective hemostasis and supports the wound healing process [34,35]. PBM cells are a popular model for evaluating the biological effects of new chemical substances due to their accessibility and ease of isolation [36,37,38]. Assessing cell viability is a fundamental step in this process [39]. The resazurin reduction assay is a widely employed method for determining cell viability [40]. The plasmid relaxation assay is commonly used to investigate the direct interaction between a compound and DNA. This method analyzes changes in DNA conformation caused by the compound’s interaction with plasmid DNA [33,41]. 

As the continuation of our research program directed on biologically active polymer-metal materials with potent medical application [42,43,44,45,46,47,48,49,50,51], we proposed the synthesis of cellulose nonwoven fabric charged with metallic copper core (CNW-Cu^0^) and covered with calcium alginate (ALG^−^Ca^2+^) layer, namely CNW-Cu^0^/Alg^−^Ca^2+^ materials.

The comparison of this composite with other representative dressing materials based on alginates is presented in Table 1.

In this study, we applied these established methodologies to examine the biological properties of CNW-Cu^0^/Alg^−^Ca^2+^ materials using PBM cells and plasmid DNA in vitro. This publication details the modification processes that build upon our previous research [51]. Cellulose nonwoven fabric, coated with a stable copper layer, underwent a double dip-coating procedure. Initially, sodium alginate was applied to the fabric, followed by immersion in calcium chloride solutions of two different concentrations. The objective of the study was to evaluate the impact of these modified materials on the blood plasma coagulation process, a critical factor for early wound treatment. Standard diagnostic assays, including activated partial thromboplastin time (aPTT) and prothrombin time (PT), were employed to assess the effects of these composites. The results demonstrated promising hemostatic properties, suggesting potential applications in medical dressings, particularly in regenerative medicine and hemostasis. A further component of our biological research focused on evaluating the impact of CNW-Cu^0^/Alg^−^Ca^2+^ materials on the viability of PBM cells, employed as a representative model of normal human cells. To explore the direct interactions between these materials and DNA, we executed the plasmid relaxation assay. This publication provides a comprehensive account of the research findings, including detailed structural characterization and chemical analysis of the modified materials.

## 2. Results and Discussion

### 2.1. Methodology for the Preparation of Composite Materials

The scheme illustrating the transformation of cellulose (CNW) into the final CNW-Cu^0^/ALG^−^Ca^2+^ is presented in Figure 1. 

Thus, the copper sputter cellulose samples (CNW-Cu^0^) (synthesized according to [51] (Figure 1(1)) were immersed in a 0.5% sodium alginate solution for a duration of one minute, ensuring a comprehensive coating process (Figure 1(2): CNW-Cu^0^ → CNW-Cu^0^/ALG^−^Na^+^). In the subsequent stage, the samples were dipped in calcium chloride solutions with concentrations of 5% or 10% (Figure 1(3): CNW-Cu^0^/ALG^−^Na^+^ → CNW-Cu^0^/ALG^−^Ca^2+^). Each immersion also lasted one minute. After completing the dip-coating, the samples were subjected to solvent evaporation and then dried at a temperature of 40 °C until they reached a constant weight. Table 2 outlines the abbreviations of the samples and the corresponding experimental conditions, providing a comprehensive overview of the preparation techniques applied in this research.

### 2.2. Determination of Metal Content

**Figure 2:**
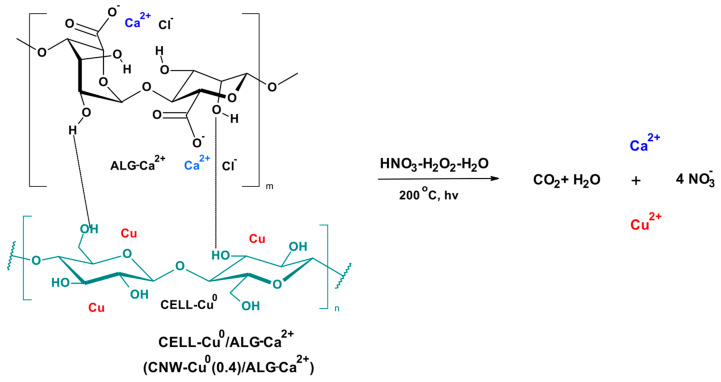
The sample degradation scheme prior to metal determination. The results were listed in Table 3.

The metals determination in prepared materials was carried out after sample digestion. This proceeds according to the reaction scheme presented in Figure 2. 

The analysis of calcium concentration in samples is fundamental for investigating both chemical and biological processes. Precisely determining the impact of calcium concentration on alginate cross-linking and blood coagulation is vital for advancing relevant technologies. In studies involving alginate and calcium chloride, the objective was to elucidate how varying concentrations of calcium chloride influence alginate cross-linking, a process critical in the fields of biotechnology and materials science. Additionally, accurate quantification of calcium levels is crucial for comprehending its role in blood coagulation, which holds significant implications for clinical and diagnostic practices.

**Table 3:** Metal concentrations in the analyzed samples.

Samples Investigated	Concentrations ^a^				Sample Codes ^b^
	Cu^0^		Ca^2+^		
	[g/kg] ^a^	[mol/kg] ^c^	[g/kg] ^a^	[mol/kg] ^d^	
CNW-Cu^0^	27.67	0.44	─		CNW-Cu^0^(0.4)
CNW-Cu^0^/ALG^−^Na^+^	27.25	0.43	─		CNW-Cu^0^(0.4)/ALG^−^Na^+^
CNW-Cu^0^/ALG^−^Na^+^,Ca^2+^-1	26.93	0.42	56.00	1.40	CNW-Cu^0^(0.4)/ALG^−^Ca(1.4)
CNW-Cu^0^/ALG^−^Na^+^,Ca^2+^-2	27.18	0.43	87.00	2.17	CNW-Cu^0^(0.4)/ALG^−^Ca(2.2)

^a^ Determinations were in triplicate. ^b^ These codes will be applied consecutively in the text. ^c^ Calculated molal concentrations for copper molecular mass 63.55 g/mol. ^d^ Calculated molal concentrations for calcium molecular mass 40.08 g/mol. All results are rounded to second decimal place; in Sample Codes the metal concentrations are rounded to first decimal place.

The application of calcium chloride solution to the samples significantly enhances the calcium content, as evidenced by an increase in concentration from 56 g/kg (1.4 molal) to 87 g/kg (2.17 molal) when using 5% and 10% solutions, respectively. Conversely, the copper concentration in the samples exhibits a slight decrease after each treatment step, suggesting a minimal impact of these processes on its content. The proportional rise in calcium content relative to the concentration of the applied solution indicates efficient saturation of the material with calcium ions.

### 2.3. Optical Microscopy Combined with Elemental Analysis Using Laser-Induced Breakdown Spectroscopy (LIBS)

#### 2.3.1. Optical Microscopy Analysis

**Figure 3:**
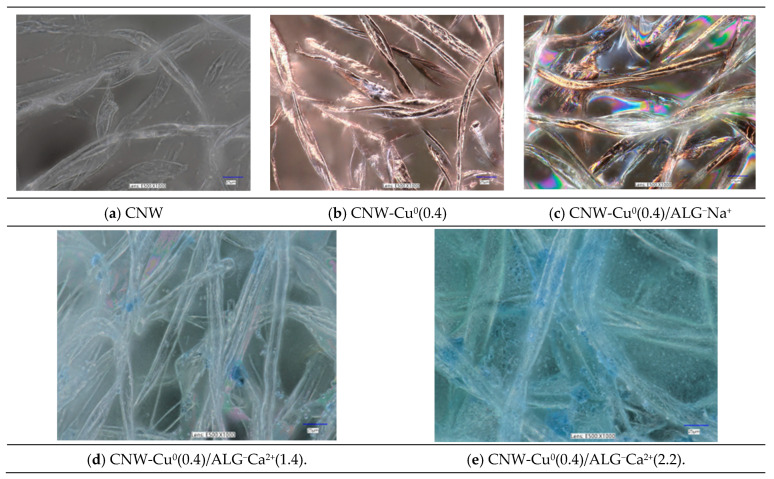
Optical microscopic images of samples: CNW (**a**); CNW-Cu^0^(0.4) (**b**); CNW-Cu^0^(0.4)/ALG^−^Na^+^ (**c**); CNW-Cu^0^(0.4)/ALG^−^Ca^2+^(1.4) (**d**); and CNW-Cu^0^(0.4)/ALG^−^Ca^2+^(2.2) (**e**). Images were recorded for magnification 1000×. The full sets of images taken for magnifications: 100×; 500× and 1000× are listed in the Figure S1 in Supplementary Materials.

The images presented microscopic views of various cellulose nanofiber samples: CNW; CNW-Cu^0^(0.4); CNW-Cu^0^(0.4)/ALG^−^Na^+^; CNW-Cu^0^(0.4)/ALG^−^Ca^2+^(1.4); and CNW-Cu^0^(0.4)/ALG^−^Ca^2+^(2.2). Each sample was examined under a microscope with magnifications 1000×, facilitating a detailed analysis of their structural and material characteristics (Figure 3).

The initial sample, CNW (Figure 3a), displays the fundamental structure of cellulose nanofibers. The microscopy images reveal slender, elongated fibers arranged in a seemingly random fashion. The structure is non-uniform, exhibiting various bends and density variations. These fibers are noted for their robust mechanical strength and high specific surface area.

The second sample, CNW-Cu^0^(0.4) (Figure 3b), consists of cellulose nanofibers modified with copper. The fiber structure remains similar to that of the CNW sample, but the images reveal additional bright reflections, potentially indicating the presence of copper. The fibers retain their irregular bends and localized densifications, but the incorporation of copper imparts new properties. Copper may enhance the antimicrobial characteristics of the fibers, thereby broadening their potential applications in the medical field. 

The third sample, CNW-Cu^0^(0.4)/ALG^−^Na^+^ (Figure 3c), incorporates both copper and alginate. The images display pronounced color reflections, indicating a more complex material composition. The fibers appear more tightly interwoven, and the additional colors may result from the interaction of light with the various components of the sample. The inclusion of alginate, a natural polysaccharide, may enhance the mechanical strength and bioactivity of the fibers, making this sample particularly promising for biomedical applications such as wound dressings.

Immersion of fibers in a calcium chloride solution induces cross-linking of alginate (CNW-Cu^0^(0.4)/ALG^−^Na^+^→CNW-Cu^0^(0.4)/ALG^−^Ca^2+^). Calcium chloride interacts with the alginate to form a polymeric network. This cross-linking process causes the alginate to become more compact and less transparent, potentially affecting the visibility of any copper metallic coating present, as illustrated in Figure 3d,e. The reaction with calcium chloride imparts a whitish hue to the alginate and reduces its transparency. Consequently, this change can result in color shifts, such as the emergence of light blue or gray tones. Additionally, the presence of calcium chloride may trigger various chemical reactions with copper, such as oxidation or complex formation, which can further alter its appearance. For example, the formation of copper compounds (e.g., copper salts) may influence the observed color shades. 

The samples, CNW-Cu^0^(0.4)/ALG^−^Ca^2+^(1.4) (Figure 3d) and CNW-Cu^0^(0.4)/ALG^−^Ca^2+^(2.2) (Figure 3e), incorporate both copper and alginate. The images display pronounced color reflections, indicating a more complex material composition. The fibers appear more tightly interwoven, and the additional colors may result from the interaction of light with the various components of the sample. The inclusion of alginate, a natural polysaccharide, may enhance the mechanical strength and bioactivity of the fibers, making this sample particularly promising for biomedical applications such as wound dressings. 

Immersion of fibers in a calcium chloride solution induces cross-linking of alginate. Calcium chloride interacts with the alginate to form a polymeric network. This cross-linking process causes the alginate to become more compact and less transparent, potentially affecting the visibility of any copper metallic coating present, as illustrated in Figure 3d,e. The reaction with calcium chloride imparts a whitish hue to the alginate and reduces its transparency. Consequently, this change can result in color shifts, such as the emergence of light blue or gray tones. Additionally, the presence of calcium chloride may trigger various chemical reactions with copper, such as oxidation or complex formation, which can further alter its appearance. For example, the formation of copper compounds (e.g., copper salts) may influence the observed color shades. 

#### 2.3.2. Elemental Analysis Using Laser-Induced Breakdown Spectroscopy (LIBS) with a Digital Microscope

Elemental analysis of the samples investigated using digital microscopy and Laser-Induced Breakdown Spectroscopy (LIBS) is presented in Table 4. The appropriate base figures are listed in the Supplementary Materials (Figure S2).

Analysis of the chemical composition of various modifications of cellulose nonwoven fabric (CNW) reveals significant changes in elemental content resulting from the applied processes. Initially, the basic cellulose nonwoven fabric primarily comprises oxygen (51.4%), carbon (40.2%), and hydrogen (8.4%). These values reflect the intrinsic chemical composition of cellulose, characterized by a predominance of these three elements.

Following magnetron sputtering with copper, the nonwoven fabric (NWC-Cu^0^(0.4)) exhibits a marked reduction in oxygen, carbon, and hydrogen content, accompanied by the introduction of copper at a concentration of 23.4%. This process incorporates copper into the fabric structure, partially substituting the original elements. These observations indicate that copper effectively deposits onto the fabric surface, thereby altering its chemical composition.

Further modification through immersion in a sodium alginate solution (CNW-Cu^0^(0.4)/ALG^−^Na^+^) induces additional changes in the chemical composition. The copper content increases to 33.9%, sodium is introduced at 0.9%, while the levels of carbon and hydrogen continue to decrease. This suggests that sodium alginate interacts with copper, forming complexes that alter the elemental ratios in the material.

The final modification through dip-coating in calcium chloride solution (CNW-Cu^0^(0.4)/ALG^−^Ca^2+^(1.4) and CNW-Cu^0^(0.4)/ALG^−^Ca^2+^(2.2)) results in further significant alterations. Immersion in a 5% CaCl_2_ solution leads to a marked increase in hydrogen, which may be attributed to measurement inaccuracies, and a reduction in copper content. The introduction of calcium from the solution is evident. Conversely, immersion in a 10% CaCl_2_ solution results in a notable rise in both oxygen and calcium content, while carbon, hydrogen, and copper levels decrease.

These modifications suggest that the dip-coating process in calcium chloride solution significantly influences the elemental distribution, potentially affecting the physicochemical properties of the final material. Each modification step introduces new elements and adjusts the ratios of existing ones, facilitating the development of materials with tailored properties for specific applications.

### 2.4. Scanning Electron Microscopy and Elemental Analysis (EDS)

#### 2.4.1. Scanning Electron Microscopy

**Figure 4:**
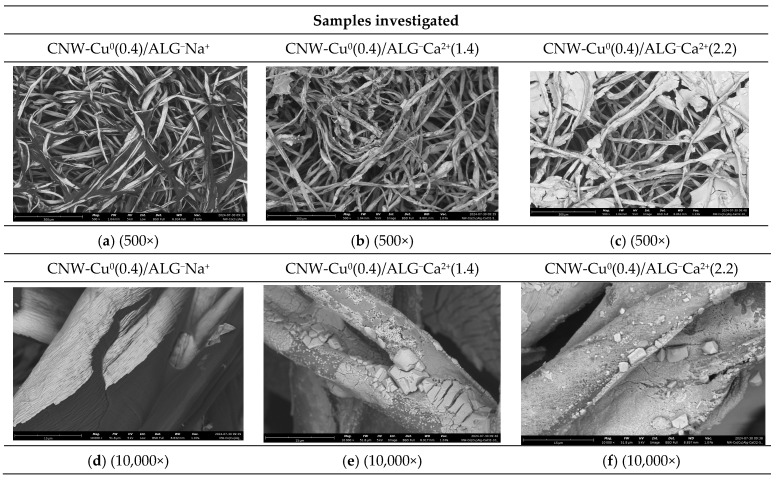
Scanning electron microscope (SEM) images of samples: CNW-Cu^0^(0.4)/ALG^−^Na^+^ (**a**,**d**); CNW-Cu^0^(0.4)/ALG^−^Ca^2+^(1.4) (**b**,**e**); and CNW-Cu^0^(0.4)/ALG^−^Ca^2+^(2.2) (**c**,**f**). Images were taken for magnification 500× and 10,000.

Scanning electron microscope (SEM) images of samples: CNW-Cu^0^(0.4)/ALG^−^Na^+^; CNW-Cu^0^(0.4)/ALG^−^Ca^2+^(1.4); and CNW-Cu^0^(0.4)/ALG^−^Ca^2+^(2.2) recorded at magnifications 500× and 10,000× are presented in Figure 4. The full sets of images taken for magnifications: 500×; 5000× and 10,000× are listed in the Supplementary Materials (Figure S3).

The examination of SEM images for the CNW-Cu^0^(0.4)/ALG^−^Na^+^; CNW-Cu^0^(0.4)/ALG^−^Ca^2+^(1.4); and CNW-Cu^0^(0.4)/ALG^−^Ca^2+^(2.2) samples reveals marked differences in their microstructural characteristics, contingent upon the specific modification processes employed (Figure 4). The CNW-Cu^0^(0.4)/ALG^−^Na^+^ sample, which consists of a cellulose nonwoven sputtered with copper and subsequently modified with sodium alginate, exhibits a loosely arranged, porous fiber network. At lower magnifications (500×), the fibers display a relatively smooth surface with minor irregularities, indicative of a homogeneous alginate coating across the fiber surface. As the magnification increases (5000× and 10,000×), more intricate features become visible, including fine cracks and localized thickenings. These features likely arise from the interactions between the alginate and cellulose fiber surfaces, as well as the effects of the copper sputtering procedure.

The CNW-Cu^0^(0.4)/ALG^−^Ca^2+^(1.4) sample, which was immersed in a 5% calcium chloride solution, exhibits distinct structural alterations. At 500× magnification, the fibers appear more densely packed, suggesting that the immersion in the CaCl₂ solution induced fiber stiffening and led to the formation of visible agglomerates on the fiber surfaces. Upon closer examination at 5000× and 10,000× magnifications, the fiber structure takes on a more granular appearance, with noticeable deposits of calcium salts forming irregular patterns. These modifications are attributed to the reaction between sodium alginate and calcium ions, resulting in the gelation of alginate and the subsequent development of a gel network on the fiber surface.

The CNW-Cu^0^(0.4)/ALG^−^Ca^2+^(2.2) sample, after immersion in a 10% calcium chloride solution, exhibits a fiber structure that is notably denser and more compact compared to the CNW-Cu^0^(0.4)/ALG^−^Ca^2+^(1.4) sample. The presence of larger agglomerates on the surface indicates a more pronounced impact of the CaCl₂ solution on the alginate matrix. At higher magnifications (5000× and 10,000×), substantial deposits of calcium salts are evident, extensively covering the fiber surface. This suggests that the increased calcium chloride concentration enhances the cross-linking of alginate, leading to a thicker and more irregular coating on the fibers.

The modification of CNW-Cu^0^(0.4)/ALG^−^Na^+^ through immersion in calcium chloride solutions notably impacts the material’s microstructure. An increase in the CaCl₂ solution concentration leads to observable changes in the fiber surface morphology. Sodium alginate reacts with calcium ions to form a gel network that encapsulates the cellulose fibers, resulting in thicker and more irregular surface structures. With a higher concentration of CaCl₂ (10%), there is a more pronounced cross-linking of the alginate, which produces larger agglomerates and increased calcium salt deposits on the fibers. This suggests that the mechanical and physicochemical properties of the material can be effectively controlled by modulating the alginate cross-linking process. Such modifications are particularly relevant for biomaterial applications, where precise control over material cross-linking, porosity, and surface roughness is essential.

#### 2.4.2. Elemental Analysis (EDS)

Elemental analysis of the samples investigated by Energy Dispersive X-ray Spectroscopy (EDS) is presented in Table 5. The appropriate base figures are listed in Figure S4 in the Supplementary Materials.

Analysis of Energy Dispersive X-ray Spectroscopy (EDS) data for various materials—CNW-Cu^0^(0.4)/ALG^−^Na^+^; CNW-Cu^0^(0.4)/ALG^−^Ca^2+^(1.4), and CNW-Cu^0^(0.4)/ALG^−^Ca^2+^(2.2), provides insights into how chemical modifications impact the materials’ chemical composition and properties (Table 5).

The high concentrations of copper, along with substantial amounts of carbon and oxygen, underscore the significant role of alginate and the presence of sputtered copper within the nonwoven matrix. Following immersion of CNW-Cu^0^(0.4)/ALG^−^Ca^2+^ in a 5% calcium chloride solution (CNW-Cu^0^(0.4)/ALG^−^Ca^2+^(1.4), alterations in the chemical composition are observed. Specifically, the carbon content decreases, which may indicate a reduction or removal of alginate due to interaction with the calcium chloride. An increase in chlorine and calcium content relative to CNW-Cu^0^(0.4)/Alg^−^Na^+^ suggests ion exchange or the incorporation of calcium chloride into the material. Additionally, the copper content decreases, potentially due to chemical interactions with the solution.

Immersion of CNW-Cu^0^/ALG^−^Na^+^ in a 10% calcium chloride solution (CNW-Cu^0^(0.4)/ALG^−^Na^+^→CNW-Cu^0^(0.4)/ALG^−^Ca^2+^(1.4)) results in additional changes to the chemical composition. The carbon content in CNW-Cu^0^(0.4)/ALG^−^Ca^2+^(2.2) is elevated relative to CNW-Cu^0^(0.4)/ALG^−^Ca^2+^(1.4) but remains lower than that in the original CNW-Cu^0^/ALG^−^Na^+^. The chlorine content shows a further increase compared to CNW-Cu^0^(0.4)/ALG^−^Ca^2+^(1.4), while the calcium content stabilizes. The copper content in CNW-Cu^0^(0.4)/ALG^−^Ca^2+^(2.2) is comparable to that in CNW-Cu^0^(0.4)/ALG^−^Ca^2+^(1.4).

### 2.5. Surface Characteristics and Pore Volume in CNW-Cu^0^/ALG^−^Ca^2+^ Samples

Surface area is one of the key physical properties for porous materials (as CNW-Cu^0^/ALG^−^Ca^2+^) since chemical reactions, such as molecular exchange, occur near or on the surface [63,64]. Among the surface area determination methods, the Brunauer-Emmett-Teller (BET) method, which determines the amount of adsorbed nitrogen monolayer at 77 K, becomes the most popular [65,66,67,68,69].

This method we applied for surface area characterization of CNW-Cu^0^/ALG^−^Ca^2+^ materials synthesized.

**Figure 5:**
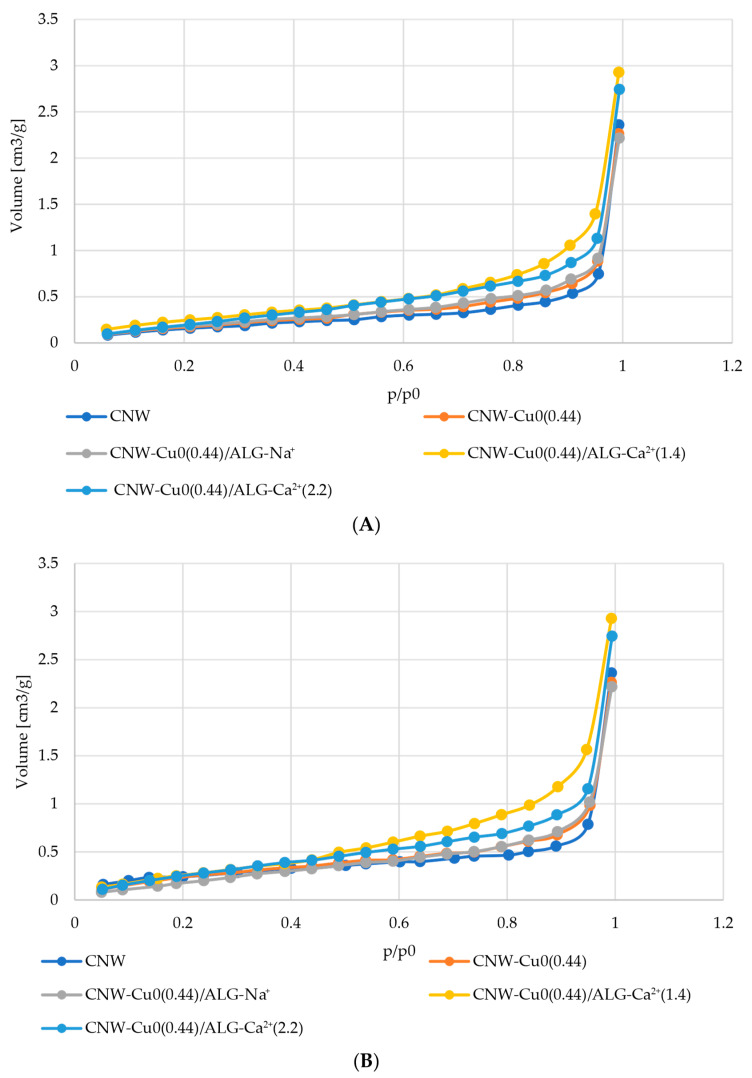
(**A**) Obtained N_2_ adsorption isotherms for NW; CNW-Cu^0^(0.44); CNW-Cu^0^(0.44)/ALG^−^Na^+^; CNW-Cu^0^(0.44)/ALG^−^Ca^+2^(1.4); CNW-Cu^0^(0.44)/ALG^−^Ca^+2^(2.2) samples. (**B**) Obtained N_2_ desorption isotherms for NW; CNW-Cu^0^(0.44); CNW-Cu^0^(0.44)/ALG^−^Na^+^; CNW-Cu^0^(0.44)/ALG^−^Ca^+2^(1.4); CNW-Cu^0^(0.44)/ALG^−^Ca^+2^(2.2) samples.

The nitrogen adsorption and desorption isotherms for CNW, CNW-Cu^0^(0.44), CNW-Cu^0^(0.44)/ALG^−^Na^+^, CNW-Cu^0^(0.44)/ALG^−^Ca^+2^(1.4), and CNW-Cu^0^(0.44)/ALG^−^Ca^+2^(2.2) samples are depicted in the collective Figure 5. Individual adsorption-desorption isotherms for the above compounds are listed in the Supplementary Materials (Figure S5).

The dip-coating modification of copper-containing cellulose nonwovens (CNW-Cu^0^), involving sequential immersion in a polysaccharide solution followed by calcium chloride solutions, induces significant alterations in their porous structure. The initial immersion in the alginate solution results in a reduction of total pore volume (TPV) and an increase in specific surface area (SSA), attributed to the polysaccharide filling the pores and creating a more complex surface. Subsequent immersions in calcium chloride solutions lead to an increase in TPV and a further rise in SSA, indicating the formation or enlargement of pores and an expansion of the accessible surface area through alginate cross-linking. Consequently, the final properties of the material are determined by the sequence of modifications and the concentrations of the applied substances, allowing for precise tuning of the material’s porous structure (Table 6).

The nitrogen adsorption-desorption isotherms depicted in Figure 5 align with type III isotherms as classified by IUPAC, characteristic of physical adsorption. This pattern reveals limited interaction between the adsorbent and the adsorbate, resulting in the accumulation of molecules mainly around favorable sites on non-porous or macroporous surfaces. The presence of such isotherms indicates that multilayer adsorption processes are prevalent throughout the entire pressure range [70,71,72].

Furthermore, the presence of a hysteresis loop suggests that the samples contain non-porous or macroporous structures. This loop reveals differences between adsorption and desorption processes, which is typical of such structures. Consequently, the samples are likely to have large, irregular pores or areas with heterogeneous porosity [73,74,75,76].

We observe that as pressure increases, the quantity of adsorbate rises exponentially, though the rate of this increase is constrained, particularly at lower relative pressures. As the relative pressure approaches values close to p/p₀ = 1, there is a marked increase in the amount of nitrogen adsorbed.

This significant rise may be attributed to the initial diffusion of nitrogen molecules into micropores at lower pressures, followed by adsorption onto a monolayer and subsequent layers at higher pressures [48]. The hysteresis loop’s shape, resembling type H3, suggests the presence of pores with a slit-like configuration [77,78,79,80]. Results from our research indicate that slit-shaped pores can retain a portion of the adsorbate at lower pressures, leading to a slower initial increase in its quantity. As pressure rises and approaches saturation, the amount of adsorbate increases significantly, which is characteristic of such pores.

### 2.6. Biological Invetigations

**Figure 6:**
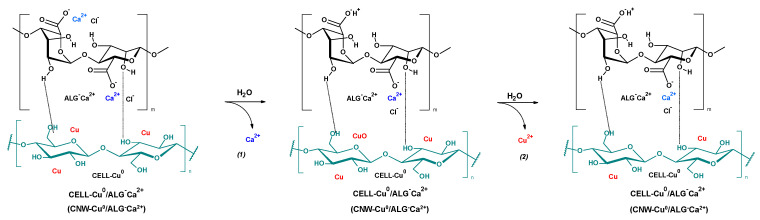
The putative mechanism of release of calcium and copper ions—agents responsible for biological activity of CNW-Cu^0^(0.44)/ALG^−^Ca^+2^ composites.

The release of calcium and copper ions from the CNW-Cu^0^(0.44)/ALG^−^Ca^+2^ composites (Figure 6) presents the main factor of their biocidal activity and hemostatic activity.

The investigations confirmed hemostetic and biocidal activity of CNW-Cu^0^(0.44)/ALG^−^Ca^+2^ composites are presented below.

#### 2.6.1. Measurement of Activated Partial Thromboplastin Time (aPTT) and Prothrombin Time (PT)

**Figure 7:**
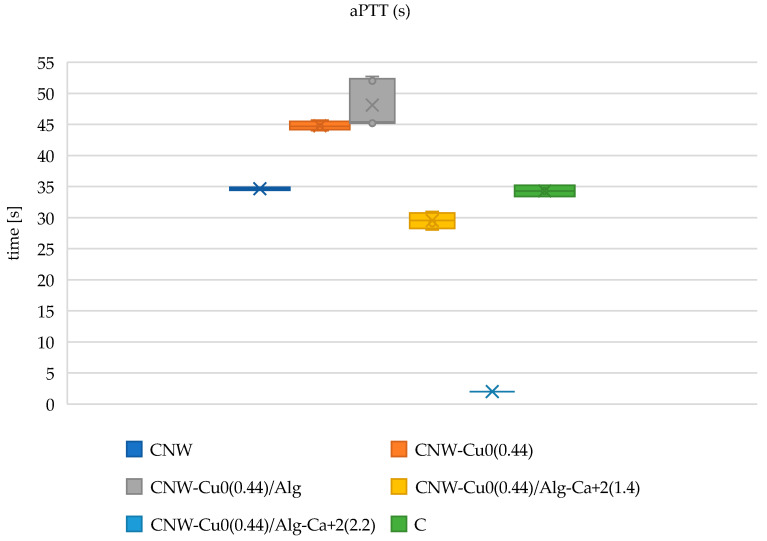
The influence of the tested copper-coated cellulose nonwoven fabrics on aPTT: CNW; CNW-Cu^0^(0.44); CNW-Cu^0^(0.44)/Alg; CNW-Cu^0^(0.44)/Alg^−^Ca^2+^(1.4); CNW-Cu^0^(0.44)/Alg^−^Ca^2+^(2.2) samples, and a plasma control (C). The data is represented with the mean (×), median (horizontal line), range (bars), and interquartile range (box).

**Figure 8:**
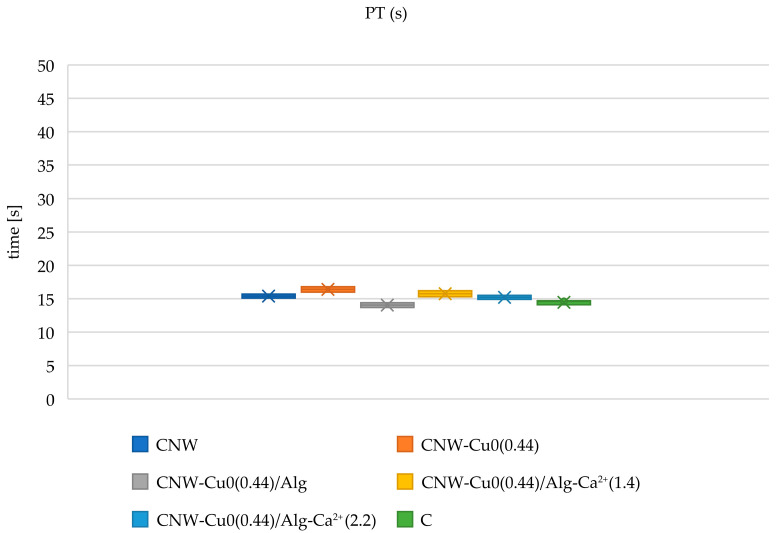
The influence of the tested copper-coated cellulose nonwoven fabrics on PT: CNW; CNW-Cu^0^(0.44); CNW-Cu^0^(0.44)/Alg; CNW-Cu^0^(0.44)/Alg^−^Ca^2+^(1.4); CNW-Cu^0^(0.44)/Alg^−^Ca^2+^(2.2) samples, and a plasma control (C). The data is represented with the mean (×), median (horizontal line), range (bars), and interquartile range (box).

The activated partial thromboplastin time (aPTT) and prothrombin time (PT) measurements for various modified cellulose nonwoven fabrics are displayed in Figure 7 and Figure 8. aPTT values provide insights into the intrinsic coagulation pathway, while PT values evaluate the extrinsic pathway. The data indicate that the modifications applied to these materials exert a range of effects on hemostatic processes, potentially due to their distinct chemical characteristics and the specific substances incorporated during modification.

As a biochemically inert material, cellulose nonwoven fabric does not actively affect blood clotting mechanisms. The polysaccharide composition of cellulose lacks direct interactions with clotting factors and does not alter their activities. Therefore, aPTT and PT measurements in samples comprising pure cellulose remain within the standard range, as cellulose does not interfere with the normal hemostatic process.

Following the sputtering of cellulose nonwoven fabric with copper (CNW-Cu^0^(0.44)), an increase in activated partial thromboplastin time (aPTT) is noted, indicating that copper might affect the intrinsic coagulation pathway. The hypothesis proposed suggests that copper interacts with key contact factors such as factors XI, XII, and HK, which are vital for the activation of this pathway [81]. This interaction may decrease the levels of these factors in the plasma, leading to an extended aPTT. In contrast, prothrombin time (PT) analysis shows only a minor increase after copper modification, reflecting a minimal impact on the extrinsic coagulation pathway. This observation implies that copper’s influence on the extrinsic pathway is limited, potentially due to compensatory mechanisms that mitigate changes in this pathway. These findings align with earlier research that explored the effects of copper sputtering on cellulose nonwoven fabric [51].

Following the modification of the nonwoven fabric with alginate (CNW-Cu^0^(0.44)/ALG^−^Na^+^), a slight further increase in activated partial thromboplastin time (aPTT) is observed. Although alginate does not possess intrinsic hemostatic properties [19,20], it may affect coagulation by interacting with coagulation factors or forming complexes that could delay the clotting process. The changes in prothrombin time (PT) in this sample are minimal, indicating that alginate has a negligible impact on the extrinsic coagulation pathway.

In samples where the CNW-Cu^0^(0.44)/ALG^−^Na^+^ nonwoven fabric was immersed in varying concentrations of calcium chloride (CNW-Cu^0^(0.44)/ALG^−^Ca^+2^(1.4) and CNW-Cu^0^(0.44)/ALG^−^Ca^+2^(2.2)), notable differences in activated partial thromboplastin time (aPTT) and prothrombin time (PT) were observed, contingent on the calcium chloride concentration. Literature indicates that calcium ions (Ca²⁺) facilitate the transformation of soluble sodium alginate into insoluble calcium alginate through a cross-linking mechanism. In the presence of blood, Ca²⁺ functions as a procoagulant by activating platelets and influencing thrombin, thereby initiating the coagulation cascade [26,27]. The observed reduction in aPTT time in CNW-Cu^0^(0.44)/ALG^−^Na^+^ samples with increasing calcium ion concentrations (CNW-Cu^0^(0.44)/ALG^−^Ca^+2^(2.2)) is attributed to the procoagulant effects of Ca²⁺ ions. Conversely, PT remains relatively stable, suggesting that factors involved in the extrinsic coagulation pathway, such as factors II, V, VII, X, and fibrinogen, may be adsorbed by alginate, leading to a decrease in their plasma concentrations.

These findings indicate that the modified materials are biodegradable, with calcium ions serving as effective coagulation activators, thus enhancing hemostasis and reducing infection risk. This study offers significant advancements in developing innovative and effective hemostatic solutions, which are critical for applications requiring precise bleeding control and infection prevention.

#### 2.6.2. Antimicrobial Activity

**Figure 9:**
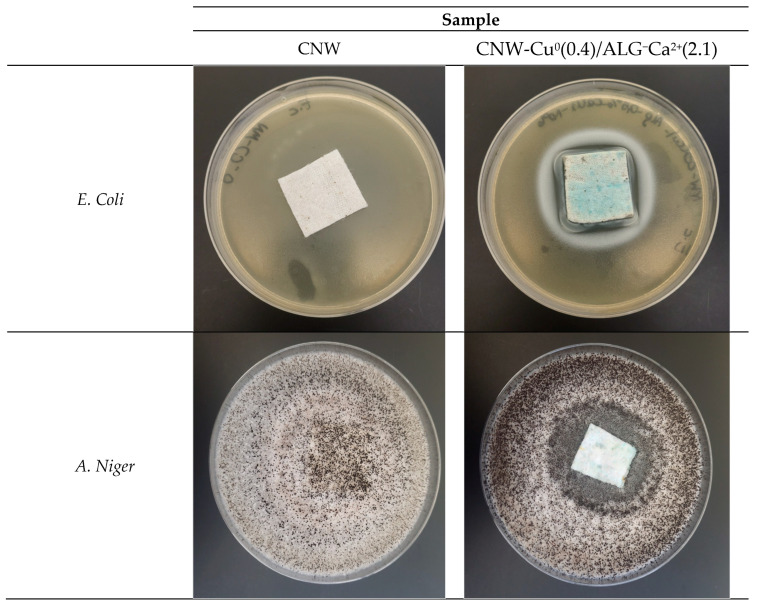
Antimicrobial activity results for CNW-Cu^0^(0.4)/ALG^−^Ca^2+^(2.2) materials on the selected bacterial and fungal strains are depicted. The images provided are for illustrative purposes.

Table 7 presents the antimicrobial efficacy of various cellulose-based samples against a range of microorganisms. The sample labeled CNW, composed exclusively of cellulose fibers, exhibited no inhibition zones across any of the tested microorganisms. This finding aligns with the expectation that pure cellulose lacks inherent antimicrobial properties and therefore does not impede microbial growth. Conversely, the CNW-Cu sample, which incorporates copper onto the cellulose fibers, demonstrated inhibition zones measuring between 2 mm and 3 mm (Figure 9). The observed antimicrobial activity is attributed to the presence of copper, which gradually undergoes corrosion (Cu^0^→CuO) and subsequently releases to the dressing environment copper ions (CNW-Cu^0^(0.4) → CNW-Cu^0^,CuO(0.4) → CNW-(Cu^0^ + Cu^2+^)).

The CNW-Cu^0^(0.4)/ALG sample, produced by immersing copper-coated cellulose in a 0.5% alginate solution, exhibited inhibition zones comparable to those observed for CNW-Cu(0.4) due to the same level of copper deposition. This suggests that the alginate cover in CNW-Cu^0^(0.4)/ALG^−^Na^+^ does not affect the antimicrobial effectiveness of the copper.

Samples CNW-Cu^0^(0.4)/ALG^−^Ca^2+^(1.4) CNW-Cu^0^(0.4)/ALG^−^Ca^2+^(2.2) demonstrate ambivalent outcomes: both samples present unchanged IZ Data for *E. Coli* (IZ = 3) and *S. Aureus* (IZ = 2), lower for *A. Niger* (IZ 2 vs. 3) and higher for *C. Globosum* (IZ 4 vs. 2).

Notably, higher calcium concentrations did not result in any changes in antimicrobial efficacy.

#### 2.6.3. Effect of Copper-Coated Cellulose Nonwoven Fabric Samples on the Viability of PBM Cells

**Figure 10:**
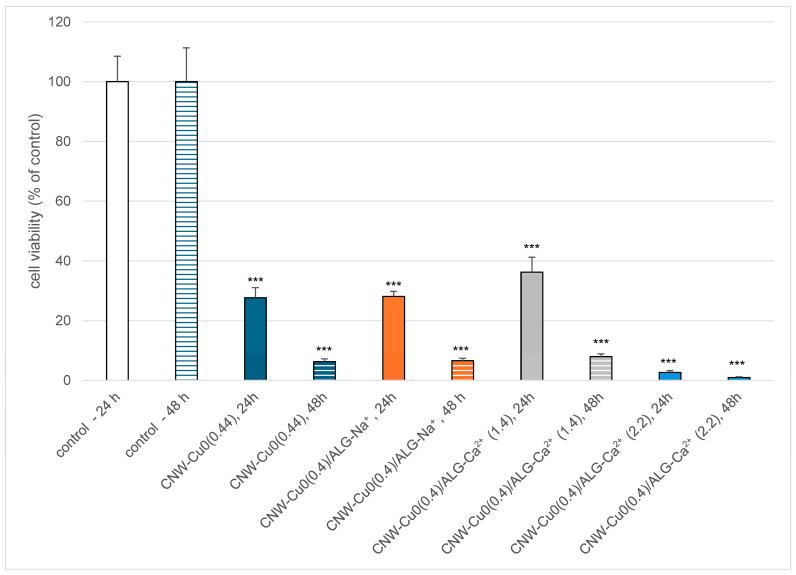
Effect of copper-coated cellulose nonwoven fabric (CNW-Cu^0^(0.44), CNW-Cu^0^(0.4)/ALG^−^Na^+^, CNW-Cu^0^(0.4)/ALG^−^Ca^2+^(1.4), CNW-Cu^0^(0.4)/ALG^−^Ca^2+^(2.2)) post-incubation mixtures on PBM cell viability after 24 and 48 h incubation. Results are presented as mean results from six repeats. Error bars denote SD; *** *p* < 0.001.

The effect of copper-coated cellulose nonwoven fabrics (CNW-Cu0(0.44), CNW-Cu0(0.4)/ALG^−^Na+, CNW-Cu0(0.4)/ALG^−^Ca^2+^(1.4), CNW-Cu0(0.4)/ALG^−^Ca^2+^(2.2)) post-incubation mixtures on PBM cell viability after 24 and 48 h incubation is presented in Figure 10.

We used the resazurin reduction assay to determine cell viability after incubation with copper-coated cellulose nonwoven fabric post-incubation mixtures. This assay is based on the application of an indicator dye to measure oxidation-reduction reactions, which principally occur in the mitochondria of live cells. The non-fluorescent dark blue dye (resazurin) becomes fluorescently pink at 570 nm and fluorescently red at neutral pH (resorufin) when reduced by metabolically active cells. We showed that incubation of PBM cells with copper-coated cellulose nonwoven fabric post-incubation mixtures decreased cell viability after 24 and 48 h incubation (Figure 10). Our results indicate the presence of cytotoxic properties of CNW-Cu^0^(0.4), CNW-Cu^0^(0.4)/ALG^−^Na^+^, CNW-Cu^0^(0.4)/ALG^−^Ca^2+^(1.4), and CNW-Cu^0^(0.4)/ALG^−^Ca^2+^(2.2) materials against PBM cells in a time-dependent manner. These results suggest that copper present in all fabrics induces a Fenton reaction [81]. During Fenton’s reaction, extremely reactive hydroxyl radicals may be formed and exhibit destructive potential towards biomolecules such as DNA, proteins, or lipids [82,83]. It should be noted that the cytotoxic effect is the most visible in the case of CNW-Cu^0^(0.4)/ALG^−^Ca^2+^(2.2). That suggests the additive effect of calcium, which increased copper’s cytotoxic potential [84].

#### 2.6.4. Effect of Copper-Coated Cellulose Nonwoven Fabric Samples on DNA

**Figure 11:**
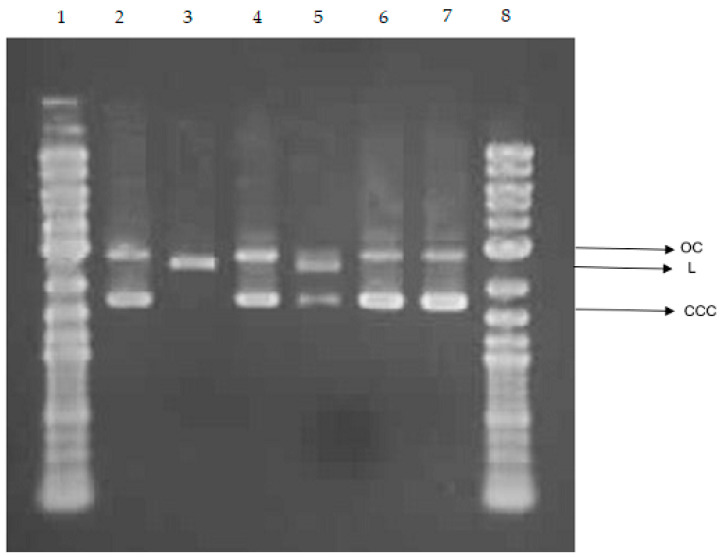
Plasmid relaxation assay. The pUC19 plasmid was incubated for 24 h (37 °C) with copper-coated cellulose nonwoven fabric post-incubation mixtures (CNW-Cu^0^(0.44), CNW-Cu^0^(0.4)/ALG^−^Na^+^, CNW-Cu^0^(0.4)/ALG^−^Ca^2+^(1.4), CNW-Cu^0^(0.4)/ALG^−^Ca^2+^(2.2)), then separated on a 1% agarose gel, stained with ethidium bromide, and visualized in UV light. Line 1—DNA ladder; line 2—pUC19 plasmid (the supercoiled form, CCC); line 3—pUC19 plasmid incubated with restrictase *Pst*I (the linear form, L); lines 4–7—pUC19 plasmid incubated with (CNW-Cu^0^(0.44), CNW-Cu^0^(0.4)/ALG^−^Na^+^, CNW-Cu^0^(0.4)/ALG^−^Ca^2+^(1.4), CNW-Cu^0^(0.4)/ALG^−^Ca^2+^(2.2)) respectively; line 8—DNA ladder. OC—the open circular form of plasmid DNA.

The effect of copper-coated cellulose nonwoven fabrics (CNW-Cu^0^(0.44), CNW-Cu^0^(0.4)/ALG^−^Na^+^, CNW-Cu^0^(0.4)/ALG^−^Ca^2+^(1.4), CNW-Cu^0^(0.4)/ALG^−^Ca^2+^(2.2)) post-incubation mixtures on DNA is presented in Figure 11.

We investigated the ability to direct interaction between CNW-Cu^0^(0.4)/ALG^−^Ca^2+^ post-incubation mixtures and DNA. For this purpose, we used the plasmid relaxation assay. Results obtained from electrophoretic mobility shift analysis (EMSA) showed that the pUC19 plasmid we isolated from the DH5α *E*. *coli* cells is presented mainly in supercoiled form (CCC). Overnight treatment at 37 °C with restrictase *Pst*I led to a linear form (L) of the plasmid. We incubated plasmid DNA with post-incubation mixtures for 24 h at 37 °C (Figure 11). In the case of CNW-Cu^0^(0.4), CNW-Cu^0^(0.4)/ALG^−^Ca^2+^(1.4), and CNW-Cu^0^(0.4)/ALG^−^Ca^2+^(2.2) post-incubation mixtures, we observe plasmid conformation such as the negative control. The incubation of pUC19 with CNW-Cu^0^(0.4)/ALG^−^Na^+^ post-incubation mixtures led to the induction of the linear form (L) of the plasmid. It suggests the ability of CNW-Cu^0^(0.4)/ALG^−^Na^+^ post-incubation mixtures to induce double-strand breaks in DNA and easier release of copper from CNW-Cu^0^(0.4)/ALG^−^Na^+^ compared to other fabrics. It was shown that copper, particularly in Cu^2+^ form, can bind to DNA [85,86,87]. Furthermore, copper ions can generate ROS through the Fenton reaction, notably producing highly reactive hydroxyl radicals [88]. 

Our findings indicate that copper-coated cellulose nonwoven materials except CNW-Cu^0^(0.4)/ALG^−^Na^+^ do not interact with DNA in vitro.

## 3. Materials and Methods

### 3.1. Materials

- In our previous research, we utilized cellulose nonwoven fabric with copper [34]. For the surface modification, we employed sodium alginate (CAS 9005-38-3, molecular weight ranging from 120,000 to 190,000 g/mol, M/G ratio of 1.56) supplied by Millipore Sigma (St. Louis, MO, USA).
- For the modification of the nonwoven copper-alginate composite surface, calcium chloride (CaCl_2_, 96%, CAS 10043-52-4) procured from Millipore Sigma (St. Louis, MO, USA) was utilized.
- The bacterial strains *Escherichia coli* (ATCC 25922) and *Staphylococcus aureus* (ATCC 6538) were obtained from Microbiologics in St. Cloud, MN, USA.
- The fungal strains *Aspergillus niger* (ATCC 6275) and *Chaetomium globosum* (ATCC 6205) were also sourced from Microbiologics in St. Cloud, MN, USA.
- We obtained lyophilized human blood plasma and clotting time assay reagents (Dia-PTT, Dia-PT, 0.025 M CaCl_2_ solution) from Diagon Kft, located in Budapest, Hungary. These products were prepared according to the manufacturer’s instructions for use with the K-3002 OPTIC coagulometers from KSELMED^®^, headquartered in Grudziądz, Poland.
- Resazurin sodium salt and molecular pure water were purchased from Sigma-Aldrich (St. Louis, MO, USA).

### 3.2. Methods

#### 3.2.1. Methodology for the Preparation of Composite Materials

The CNW-Cu^0^ nonwoven fabric, which was coated with a copper layer using the magnetron sputtering technique as detailed in the referenced publication, underwent a dip-coating process in a 0.5% (*w*/*v*) aqueous sodium alginate solution [51]. The fabric was submerged in the solution for 1 min. Subsequently, the fabric was immersed in calcium chloride solutions of varying concentrations (5% and 10% *w*/*v*) for 1 min, facilitating the ionic cross-linking of the alginate and the formation of a stable hydrogel matrix. Post-cross-linking, the fabric was thoroughly rinsed with deionized water and dried at 40 °C until constant weight, after which it was stored in a dry environment for future analysis.

#### 3.2.2. Determination of Calcium and Copper Content

The determination of copper content in the composite samples was conducted using a single-module Magnum II microwave mineralizer (Ertec, Wrocław, Poland). The digestion process occurred within a closed system, which enabled precise control over temperature and pressure. For the digestion procedure, 2.5 mL of 65% nitric acid (HNO₃) and 2.5 mL of hydrogen peroxide (H₂O₂) were added to the samples to ensure comprehensive digestion. Post-digestion, the quantification of calcium was performed using an ICP-MS 7900 (Agilent Technologies, Santa Clara, CA, USA). The calcium content was assessed employing the same analytical method. To ensure the reliability and uniformity of the data, each sample was measured twice, and the mean values were reported as the final results.

#### 3.2.3. Microscopy Analysis

Microscopic analysis was conducted using both optical and scanning electron microscopy techniques. Optical microscopy was utilized to examine and document the general morphology and structural features of the samples at lower magnification. Elemental composition was analyzed using a digital microscope equipped with Laser Induced Breakdown Spectroscopy (LIBS) technology. For a more detailed evaluation of surface characteristics, scanning electron microscopy (SEM) was employed. SEM provided high-resolution images to investigate the surface topography and finer structural details of the samples. Optical imaging was performed using a VHX-7000N digital microscope (Keyence, Japan), while SEM analysis was carried out with a Phenom ProX G6 scanning electron microscope (Thermo Fisher Scientific, Waltham, MA, USA). SEM imaging was conducted under low vacuum conditions (60 Pa) with an accelerating voltage of 15 keV. The combination of these techniques facilitated a thorough analysis of the sample characteristics.

#### 3.2.4. Evaluation of Specific Surface Area and Total Pore Volume

The specific surface area and total pore volume of the samples were determined using the Brunauer–Emmett-Teller (BET) method. This analysis was performed with an Autosorb-1 instrument (Quantachrome Instruments, Boynton Beach, FL, USA), employing nitrogen gas at 77 K as the adsorption agent. Prior to measurement, the samples were dried at 105 °C for 24 h to remove moisture. Subsequently, the samples were degassed at room temperature to ensure accurate results. Approximately 2 g of each sample were weighed and used for the BET analysis.

#### 3.2.5. Measurement of Activated Partial Thromboplastin Time (aPTT) and Prothrombin Time (PT)

Frozen and freeze-dried human plasma was reconstituted with deionized water. For assay preparation, 1 mg of the reconstituted plasma sample was combined with 200 µL of plasma. Following this, the mixture was centrifuged and incubated for 15 min at a precisely controlled temperature of 37 °C. The activated partial thromboplastin time (aPTT) was evaluated using a Dia-PTT reagent, which was composed of kaolin, cephalin, and a 0.025 M calcium chloride (CaCl₂) solution. The aPTT assays were carried out using a K-3002 OPTIC coagulometer. Each assay involved adding 50 µL of plasma and 50 µL of the Dia-PTT reagent to the coagulometer’s thermostat, which was maintained at 37 °C. After 3 min of incubation, 50 µL of the 0.025 M CaCl₂ solution was added to commence the measurement.

To evaluate prothrombin time (PT), 100 µL of the plasma sample was incubated for 2 min at a precisely controlled temperature of 37 °C. Subsequently, 100 µL of the Dia-PTT suspension was introduced to initiate the measurement. The Dia-PTT suspension comprised thromboplastin derived from rabbit brain tissue, calcium ions, and a preservative. To ensure the accuracy of the measurements, the suspension was meticulously mixed prior to each use.

#### 3.2.6. Antimicrobial Activity

The antimicrobial properties of the developed materials were evaluated according to the PN-EN ISO 20645:2006 standard [89]. This evaluation included testing against both Gram-negative (*E. coli*, ATCC 25922) and Gram-positive (*S. aureus*, ATCC 6538) bacteria using the agar diffusion method on Mueller–Hinton agar plates. Sterilized agar was poured into Petri dishes, which were then inoculated with bacterial broth cultures. After incubation, the material samples were placed on the agar. Following a 24 h incubation period at 37 °C, the diameters of the inhibition zones were measured to determine antimicrobial activity.

The antifungal properties were assessed according to the PN-EN 14119:2005 standard [90]. This assessment utilized *C. globosum* (ATCC 6205) and *A. niger* (ATCC 6275) fungi. Samples were placed on agar plates inoculated with these fungi and incubated at 29 °C for 14 days. After the incubation period, fungal growth at the interface between the agar and the sample surfaces was examined, and any visible inhibition zones were recorded. Each assay was performed in duplicate.

#### 3.2.7. PBM Cells

Peripheral blood mononuclear cells (PBM cells) were isolated from a leucocyte-buffy coat collected from the blood of healthy non-smoking donors from the Blood Bank in Lodz, Poland, as described previously [91]. The first step of isolation of PBM cells was a mix of fresh blood from buffy coats with PBS at a ratio of 1:1. In the next step, the mixture was centrifuged in a density gradient of Lymphosep (Cytogen, Zgierz, Poland) at 2200 RPM for 20 min with the lowest values of acceleration and deceleration. Then PBM cells were washed three times by centrifugation with 1% PBS. After isolation, cells were suspended in RPMI 1640 medium. The study protocol was approved by the Committee for Research on Human Subjects of the University of Lodz (17/KBBN-UŁ/III/2019).

To analyze the influence of CNW-Cu^0^(0.4)/ALG^−^Ca^2+^ composite materials on PBM cell viability, fragments of CNW-Cu^0^(0.4)/ALG^−^Ca^2+^ composite fabrics were cut into 1 cm^2^ (1 × 1 cm), seeded on a 6-well plate, and incubated with 3 mL of RPMI medium at 37 °C in 5% CO_2_ for 24 h as described previously [50]. After that, post-incubation mixtures were filtered with a 0.2 µm filter to obtain an aseptic condition. Then copper-coated cellulose nonwoven fabric post-incubation mixtures were added to PBM cells in a ratio of 1:1 to analyze their influence on cell viability.

#### 3.2.8. Cell Viability by the Resazurin Reduction Assay

The cell viability resazurin assay was performed using the method described by O’Brien et al. [92]. Resazurin salt powder was dissolved in sterile PBS buffer. Post-incubation mixtures were added to PBM cells in the count of 5 × 10^4^ and then incubated for 24 and 48 h at 37 °C in 5% CO_2_. The negative control was RPMI 1640 medium prepared in the same manner as post-incubation mixtures. Next, 10 µL of resazurin salt was added to each well, and the plates were incubated at 37 °C in 5% CO_2_ for 2 h. After that, fluorescence was measured with HT microplate reader BioTek Synergy HT (Agilent Technologies, Inc., Santa Clara, CA, USA) using λ_ex_ = 530/25 and an λ_em_ = 590/35 nm. The effects of copper-coated cellulose nonwoven fabric post-incubation mixtures were quantified as the percentage of control fluorescence.

#### 3.2.9. DNA Damage by the Plasmid Relaxation Assay

To analyze the influence of CNW-Cu^0^(0.4)/ALG^−^Ca^2+^ composite materials on plasmid DNA, fragments of CNW-Cu^0^(0.4)/ALG^−^Ca^2+^ composite fabrics were cut into 1 cm^2^ (1 × 1 cm), seeded on a 6-well plate, and incubated with 3 mL of molecular pure water at 37 °C in 5% CO_2_ for 24 h. Then, post-incubation mixtures were filtered with a 0.2 µm filter to obtain an aseptic condition. Then copper-coated cellulose nonwoven fabric post-incubation mixtures were added to DNA plasmid in a ratio of 1:1 to analyze their influence on DNA plasmid conformation.

The plasmid relaxation assay was performed similar to the procedure of Juszczak et al. [93]. The pUC19 plasmid was isolated from the DH5α *E. coli* cells with the AxyPrep Plasmid Miniprep Kit (Axygen) according to the manufacturer’s instructions. The isolated plasmid quantity and quality were determined by the A260/A280 ratio and gel electrophoresis, respectively. The native form of pUC19 exists mainly in the supercoiled form (CCC), which is characterized by a relatively high electrophoretic mobility. The plasmid was digested with the restrictase *Pst*I (New English Biolabs, Ipswich, MA, USA) to induce linear (L) form. Topological differences between CCC and L forms of the plasmid account for their different electrophoretic mobility. The plasmid at 50 ng μL^−1^ was incubated for 24 h with CNW-Cu^0^(0.4)/ALG^−^Ca^2+^ composite post-incubation mixtures. Then the samples were subjected to 1% agarose gel electrophoresis with ethidium bromide staining, visualization under UV light (302 nm), scanning by a CCD camera, and analysis with the GeneTools 4.3.9.0 by Syngene (Cambridge, UK) software. During electrophoresis, we separated 4 μL of 1 kb DNA ladder (GeneRuler 1 kb DNA ladder, Thermo Scientific, Waltham, MA, USA).

#### 3.2.10. Statistical Analysis

The data is presented as mean ± SD. The statistical analysis was conducted using the student’s two-tailed *t*-test. The differences were considered statistically significant when the *p*-value was <0.05.

## 4. Conclusions

Wound dressing materials with antibacterial and hemostatic properties present innovative solutions to challenges faced in regenerative medicine, particularly in addressing antibiotic resistance during wound care and healing. This research focuses on the design and characterization of composite materials comprising cellulose nonwoven fabric, copper, sodium alginate, and calcium ions. These CNW-Cu^0^/ALG^−^Ca^2+^ composites were synthesized through a three-step process. Initially, the cellulose nonwoven fabric was coated with copper using magnetron sputtering, as described in our earlier work. This was followed by a dip coating procedure involving sodium alginate and calcium ions.

The composites underwent chemical and structural analysis using flame atomic absorption spectrometry (FAAS) and Brunauer-Emmett-Teller (BET) surface area analysis.

The newly developed materials demonstrated in vitro antibacterial properties (CNW-Cu^0^→ CNW-Cu^2+^→ CNW + Cu^2+^) against typical Gram-negative (*Escherichia coli*) and Gram-positive (*Staphylococcus aureus*) and fungi (*Chaetomium globosum* and *Aspergillus niger*).

Additionally, the biochemical properties of the composites were evaluated concerning blood plasma clotting processes, including activated partial thromboplastin time (APTT) and prothrombin time (PT).

Results from biological assessments indicated that incorporating calcium ions positively influences blood plasma clotting (CNW-Cu^0^/ALG^−^Ca^2+^ → CNW-Cu^0^/ALG^−^ + Ca^2+^). These findings suggest that cellulose-copper-alginate-calcium nonwoven composites are viable for biomedical applications such as wound dressings, as they effectively reduce clotting time.

The copper-coated cellulose nonwoven fabric materials decreased PBM cell viability. We did not observe the ability of these materials to directly interact with DNA, except CNW-Cu^0^/ALG^−^Na^+^, which can induce double-strand breaks of DNA. Among the different variations tested, CNW-Cu^0^/ALG^−^Ca^2+^(1.4) showed the least harmful effects on PBM cells. To further understand the biological properties of the copper-coated cellulose nonwoven fabric (CNW-Cu) these materials, additional studies using various molecular biology techniques and other normal human cell types, such as skin fibroblasts, are recommended.

The significant benefits of these composites include cost-effectiveness, high availability of raw materials, and the simplicity of the manufacturing process. The combined effects of reduced blood clotting time (ALG^−^Ca^2+^) and antibacterial properties (CNW-Cu^0^) highlight the potential of these composites in wound dressings, offering a dual function in the prevention and treatment of infections.

## Figures and Tables

**Table 1:** Representative hemostatic composites based on alginates.

Composite	Core		Upper Layer		Biomedical Application	Ref.
	Polym.	Agent	Polym. (Formed) ^a^	Agent		
ALG^−^Ca^2+^, Na^+^	ALG^−^Na^+^	CaCl_2_^a^			hemostatic tamponade	[52]
ALG^−^H^+^/ALG^−^Ca^2+^	ALG^−^H^+^		ALG^−^Na^+^	CaCl_2_	hemostasis/breast cancer wounds	[53]
ALG^−^Na^+^/TiO_2_ and ZnO	ALG^−^Na^+^-F	TiO_2_ and ZnO			biocidal	[54]
ALG^−^Ca^2^ and Zn^2+^	ALG^−^Na^+^	CaCl_2_, Zn^+^Cl_2_			pre-hospital hemostasis	[55]
ALG^−^Ca^2+^/THR^+^	ALG^−^Na^+^	CaCl_2_	(ALG^−^Ca^2+^)	THR	hemostatic embolization	[56]
CTS/ALG^−^Ca^2+^	CTS	ALG^−^Na^+^	(CTS/ALG^−^Na^+^)	CaCl_2_	traumatic hemostat	[57]
CTS/ALG^−^Ca^2+^	CTS	ALG^−^Ca^2+^			wound healing	[58]
CTS^*^/ALG^−^Na^+^/ZnO	CTS	ALG^−^Na^+^	(CTS^*^/ALG^−^Na^+^)	ZnO	bandages for infected wounds	[59]
GEL/ALG^−^Ca^2+^/HA and PHHB	GEL-ALG^−^Na^+^	Ca^2+^Cl_2_	(GEL/ALG^−^Ca^2+^)	PHMB and HA-LBL	hemostasis and disinfection	[60]
SF/ALG^−^Ca^2+^	SF	ALG^−^Na^+^	(SF/ALG^−^Na^+^)	CaCl_2_	hemostasis in vitro	[61]
SF/GA/ALG^−^Ca^2+^	SF/GEL	ALG^−^Na^+^	(SF/GA/ALG^−^Na^2+^)			
CNW-Cu^0^-ALG^−^Ca^2+^	CNW-Cu^0^	ALG^−^Na^+^	(CNW-Cu^0^-ALG^−^Na^+^)	CaCl_2_	hemostatic and biocidal	This work

^a^ Formed by core treatment. Not equivalently to ALG^−^Na^+^chitosan. Abbreviations: ALG^−^H^+^—alginic acid; ALG^−^Ca^2+^—calcium alginate; ALG^−^Na^+^—sodium alginate; CTS—chitosan; GA—gelatin; HA—Hyaluronic Acid; LBL—Layer by LAYER Assembly; PHMB—Poly(Hexamethylene Biguanide)-hydrochloride; SF—silk fibroin; THR—thrombin.

**Table 2:** Experimental data for magnetron sputtering and dip-coating processes.

Parameter	Range	Mixture Components of Film-Forming Material (%)		
Gas Pressure	2.3 × 10^−3^ mbar	**Sodium Alginate Solution**	**Calcium Chloride Solutions**	
Magnetron Power	0.4 kW and 0.8 kW			
Samples Investigated	Process duration (Magnetron power)	0.5%	5%	10%
CNW-Cel	−	−	−	−
CNW-Cu^0^	32 min (0.8 kW)	−	−	−
CNW-Cu^0^/ALG^−^Na^+^	32 min (0.8 kW)	+	−	−
CNW-Cu^0^/ALG^−^Na^+^,Ca^2+^-1	32 min (0.8 kW)	+	+	−
CNW-Cu^0^/ALG^−^Na^+^,Ca^2+^-2	32 min (0.8 kW)	+	−	+

**Table 4:** Elemental analysis of the samples investigated using digital microscopy and Laser-Induced Breakdown Spectroscopy (LIBS).

Analyzed Samples	Determined Elements					
	C	H	O	Cu	Na	Ca
Cellulose ^a^	42.35	6.12	51.53			
C_6_H_10_O_5_^b^	44.45	6.22	49.34			
CNW	40.2	8.4	51.4			
CNW-Cu	26.0	5.4	45.2	23.4		
C_6_H_10_O_5_Cu ^c^	31.93	4.47	35.45	28.16		
CNW-Cu^0^(0.4)/ALG^−^Na^+^	18.5	5.1	41.6	33.9	0.9	
CNW-Cu^0^(0.4)/ALG^−^Ca^2+^(1.4)	16.9	52.3	29.3	0.2		1.3
CNW-Cu^0^(0.4)/ALG^−^Ca^2+^(2.2)	13.79	7.2	67.89	4.6		6.7

^a^ Literature data [62]. ^b^ Calculated for the molecular formula [162.1]. ^c^ Calculated for the molecular formula [225.7].

**Table 5:** Elemental analysis of the samples investigated using Energy Dispersive X-ray Spectroscopy (EDS).

	Elements									
	C		O		Ca		Cu		Cl	
	A.C.	W.C.	A.C.	W.C.	A.C.	W.C.	A.C.	W.C.	A.C.	W.C.
CNW-Cu^0^(0.4)/Alg^−^Na^+^	42.07	26.2	47.49	39.4			10.44	34.40		
CNW-Cu^0^(0.4)/Alg^−^Ca^2+^(1.4)	23.15	11.64	40.44	27.08	10.82	18.15	4.38	11.64	21.22	31.49
CNW-Cu^0^(0.4)/Alg^−^Ca^2+^ (2.2)	26.5	13.89	40.91	28.57	10.45	18.28	4.07	11.29	18.07	27.97

A.C.—Atomic Concentration: The atomic percentage is the number of atoms of that element at that weight percentage, divided by the total number of atoms in the sample multiplied by 100. W.C.—Weight Concentration: The weight percentage of an element is the weight of that element measured in the sample divided by the weight of all elements in the sample multiplied by 100. Concentration data are rounded to the second decimal place.

**Table 6:** Specific surface area and total pore volume for a sample of unmodified cellulose nonwoven fabric (CNW) and Cel-Cu materials (CNW-Cu^0^; CNW-Cu^0^/ALG; CNW-Cu^0^/ALG^−^Ca^2^).

Sample Name	Total Pore Volume (TPV)	Specific Surface Area (SSA)
	cm^3^/g	m^2^/g
CNW	3.714 × 10^−3^	5.98 × 10^−1^
CNW-Cu(0.4)	3.491 × 10^−3^	8.55 × 10^−1^
CNW-Cu(0.4)/ALG	3.438 × 10^−3^	7.40 × 10^−1^
CNW-Cu(0.4)/ALG^−^Ca^2+^(1.4)	4.254 × 10^−3^	9.79 × 10^−1^
CNW-Cu(0.4)/ALG^−^Ca^2+^(2.2)	4.541 × 10^−3^	9.97 × 10^−1^

**Table 7:** Antibacterial activity results for materials.

Sample ^a^	Average Inhibition Zone(mm)				*LIT*
	Bacteria		Fungi		
	*E.* *coli*	*Staph. aureus*	*A. niger*	*C. globosum*	
CNW	0	0	0	0	
	0	0	0	0	[51]
CNW-Cu^0^(0.2)	2	1	1	2	[51]
CNW-Cu^0^(0.4)	3	2	3	2	
CNW-Cu^0^(0.4)	3	2	3	2	[51]
CNW-Cu^0^(0.4)/ALG^−^Na^+^	3	2	3	2	
CNW-Cu^0^(0.4)/ALG^−^Ca^2+^(1)	3	2	2	4	
CNW-Cu^0^(0.4)/ALG^−^Ca^2+^(2)	3	2	2	4	
PLA-ALG^−^Na^+^	0	0	0	0	[42,45]
PLA-ALG-Cu^2+^(0.2)	3	2	3	3	[42]
PLA-ALG-Cu^2+^(1.2)	3	4	3	3	[42]
PLA-ALG-Zn^2+^(0.2)	0	>1	<1	<1	[45]
PLA-ALG-Zn^2+^(0.4)	>1	>1	<1	<1	[45]
PLA-Cu(0.2)	2	1		1	[44]
PLA-Cu(0.4)	2	1		3	[44]
WO-Cu^0^(0.1)	1	1		1	[50]
WO-Cu^0^(0.4)	3	2		1	[50]
PET-Cu(0.1)	1	1		3	[43]
PET-Cu(0.2)	2	1		3	[43]

^a^ All results are rounded to the first decimal place. Abbreviations: Fibers: CNW—cellulose non woven fabric; PLA—PolyLactic Acid; PET—PolyEthylene Terephthalate; WO—wool Bacteria: *E. coli—Escherichia coli*; *Staph. aureus—Staphylococcus aureus.* Fungi: *A. niger—Aspergillus niger*; *C. globosum— Chaetomium globosum.* Concentration of inoculum [CFU/mL]: *E. coli*: = 1.5 × 108; *S. aureus*: = 1.3 × 108; *A. Niger*: 1.8 × 106; *C. Globosum*: 2.1 × 106.

## Data Availability

The original contributions presented in the study are included in the article/Supplementary Materials, further inquiries can be directed to the corresponding author.

## Supplementary Materials

The following supporting information can be downloaded at: https://www.mdpi.com/article/10.3390/md22100436/s1, Figure S1. Optical microscopy combined with elemental analysis using laser-induced breakdown spectroscopy (LIBS); Figure S2. Elemental Analysis Using Laser-Induced Breakdown Spectroscopy (LIBS) with a Digital Microscope; Figure S3. Scanning electron microscopy and elemental analysis (EDS); Figures S4. Energy dispersive X-ray spectroscopy (EDS) experimental data for CNW-Cu^0^/Alg-Na+, CNW-Cu^0^/Alg-Ca^2+^(1.4) and CNW-Cu^0^/Alg-Ca^2+^(2.2); Figure S5. Obtained N_2_ adsorption-desorption isotherms for: (a) CNW; (b) CNW-Cu^0^(0.44); (c) CNW-Cu^0^(0.44)/ALG-Na^+^; (d) CNW-Cu^0^(0.44)/ALG-Ca^+2^(1.4); (e) CNW-Cu^0^(0.44)/ALG-Ca^+2^(2.2) samples.
