# Supplementary material for: Preparation and Biochemical Activity of Copper-Coated Cellulose Nonwoven Fabric via Magnetron Sputtering and Alginate-Calcium Ion Complexation
Source: Mar Drugs. 2024 Sep 26;22(10):436. doi: 10.3390/md22100436 (PMC11509239; doi:10.3390/md22100436)
Supplement: Supplementary file 1 [file marinedrugs-22-00436-s001.zip › marinedrugs-3195405-supplementary.pdf]

Supplementary

Preparation and Biochemical Activity of Copper-Coated Cellulose Nonwoven Fabric via Magnetron Sputtering and Alginate-Calcium Ion Complexation

Małgorzata Świerczyńska [1,2], Zdzisława Mrozińska [1], Michał Juszczak [1,3], Katarzyna Woźniak [3] and Marcin H. Kudzin [1,*]

Figure S1. Optical microscopy combined with elemental analysis using laser-induced breakdown spectroscopy (LIBS).

**Sample: CNW**		
**Magnification**		
100 x	500 x	1000 x

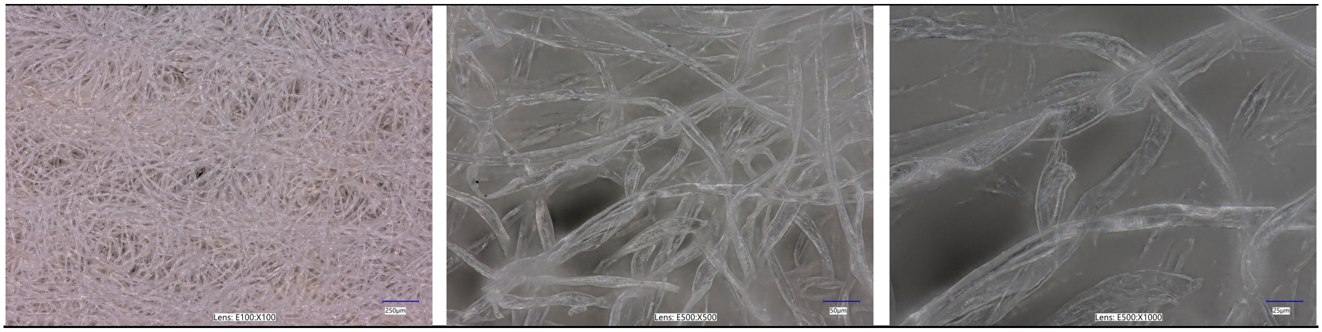

Figure S1.1. Optical microscopic images of samples before dip-coating processes, sample: CNW. Magnification: 100×; 500×; 1000×.

**Sample: CNW-Cu^0**		
**Magnification**		
100 x	500 x	1000 x

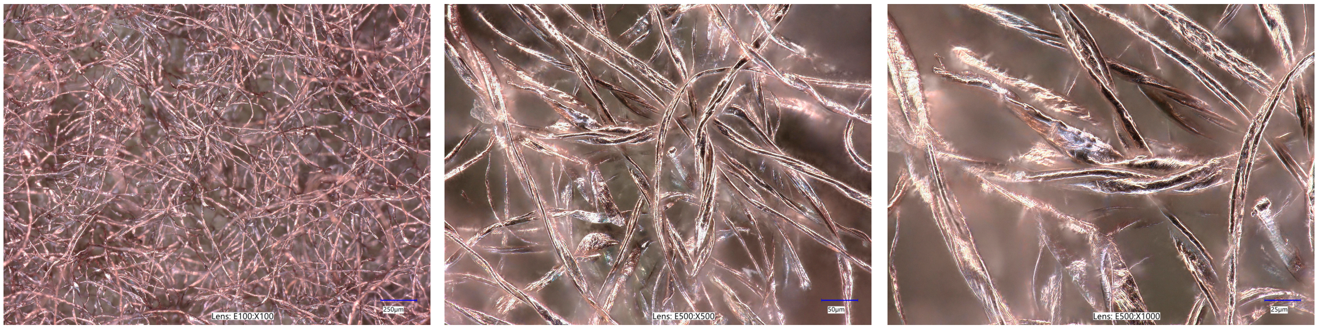

Figure S1.2. Optical microscopic images of samples after dip-coating processes, sample: CNW-Cu^0. Magnification: 100×; 500×; 1000×.

Sample: **CNW-Cu^0/Alg^-Na^+**		
**Magnification**		
100 x	500 x	1000 x

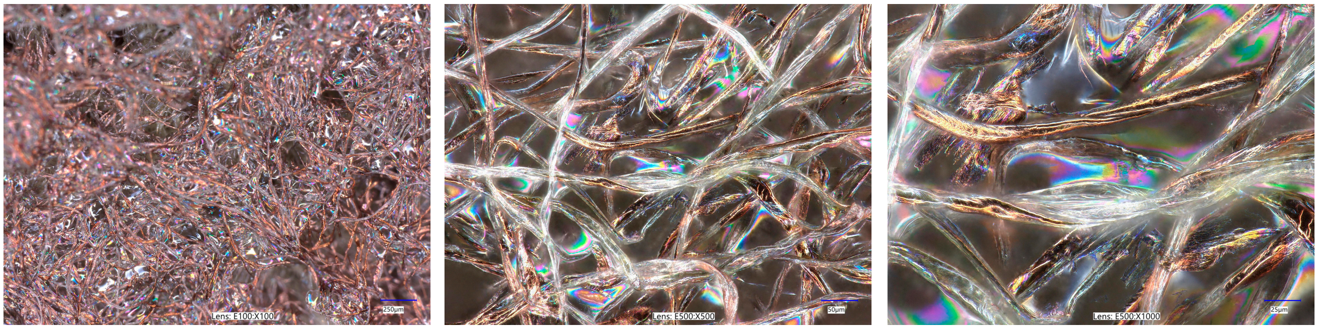

Figure S1.3. Optical microscopic images of samples after dip-coating processes, sample: **CNW-Cu^0/Alg^-Na^+**. Magnification: 100×; 500×; 1000×.

Sample: **CNW-Cu^0/Alg^-Ca^{2+}(1.4)**		
**Magnification**		
100 x	500 x	1000 x

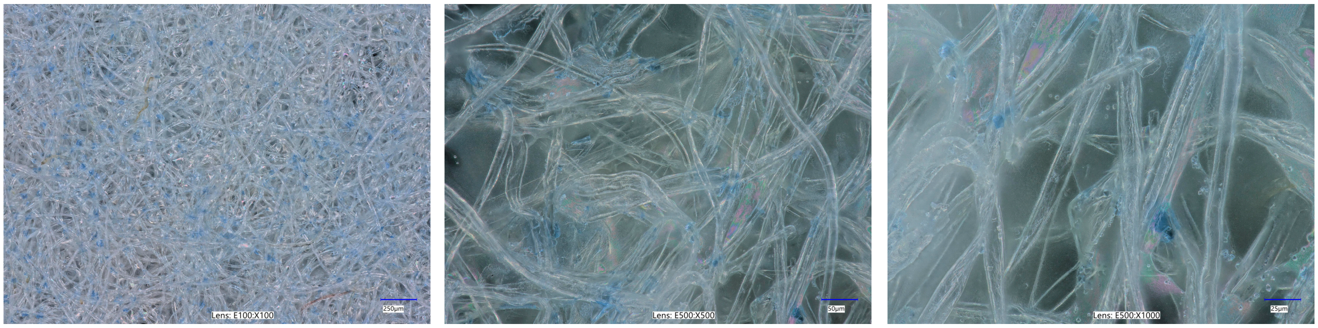

Figure S1.4. Optical microscopic images of samples after dip-coating processes, sample: **CNW-Cu^0/Alg^-Ca^{2+}(1.4)**. Magnification: 100×; 500×; 1000×.

Sample: **CNW-Cu^0/Alg^-Ca^{2+}(2.2).**		
**Magnification**		
100 x	500 x	1000 x

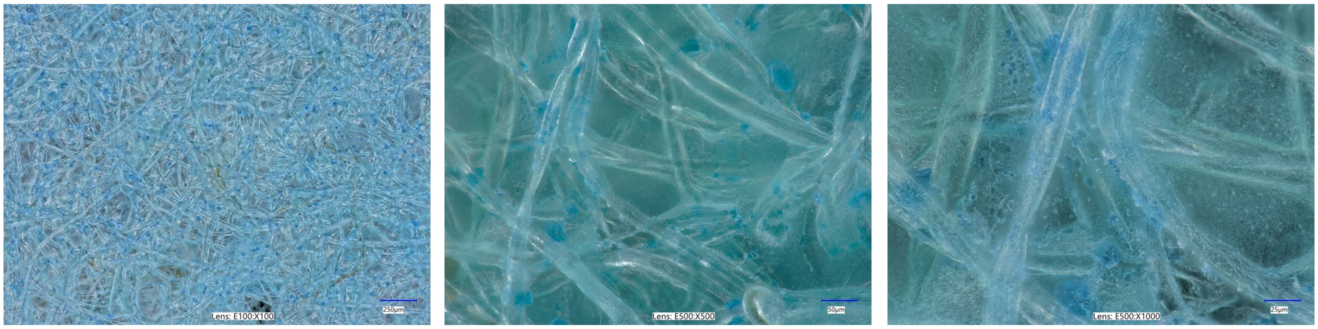

Figure S1.5. Optical microscopic images of samples after dip-coating processes, sample: **CNW-Cu^0/Alg^-Ca^{2+}(2.2)**. Magnification: 100×; 500×; 1000×.

Figure S2. Elemental Analysis Using Laser-Induced Breakdown Spectroscopy (LIBS) with a Digital Microscope

Sample: CNW

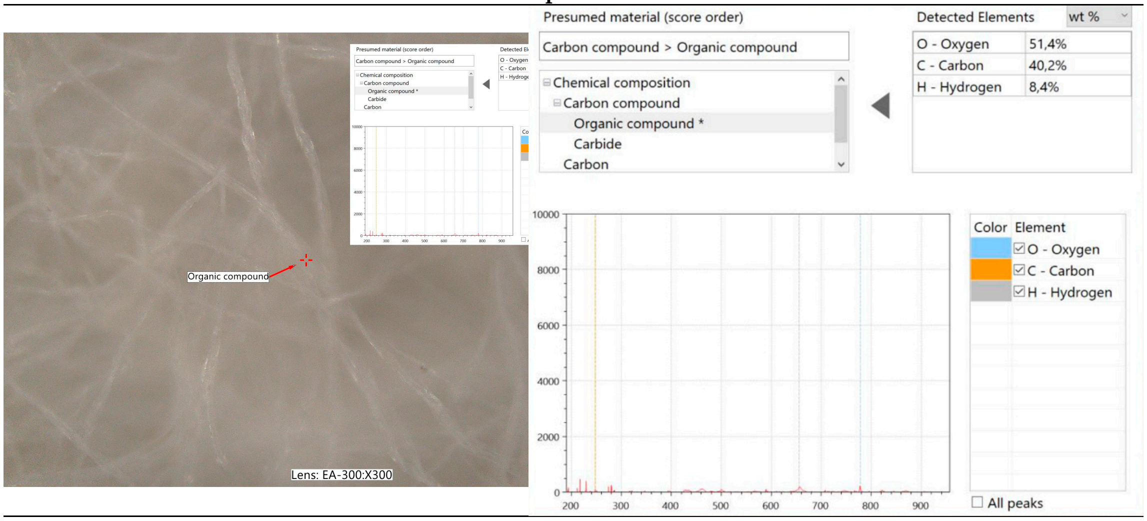

Figure S2.1. Elemental analysis of the CNW sample using digital microscopy and Laser-Induced Breakdown Spectroscopy (LIBS).

Sample: CNW-Cu^0(0.44)

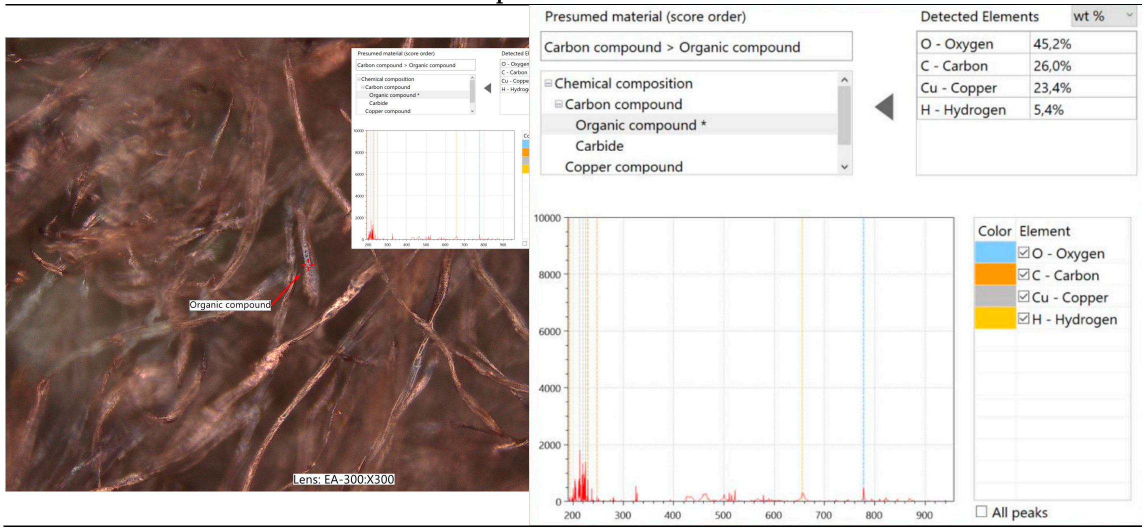

Figure S2.2. Elemental analysis of the **CNW-Cu^0(0.44)** sample using digital microscopy and Laser-Induced Breakdown Spectroscopy (LIBS).

Sample: CNW-Cu^0(0.44)/Alg^-Na^+

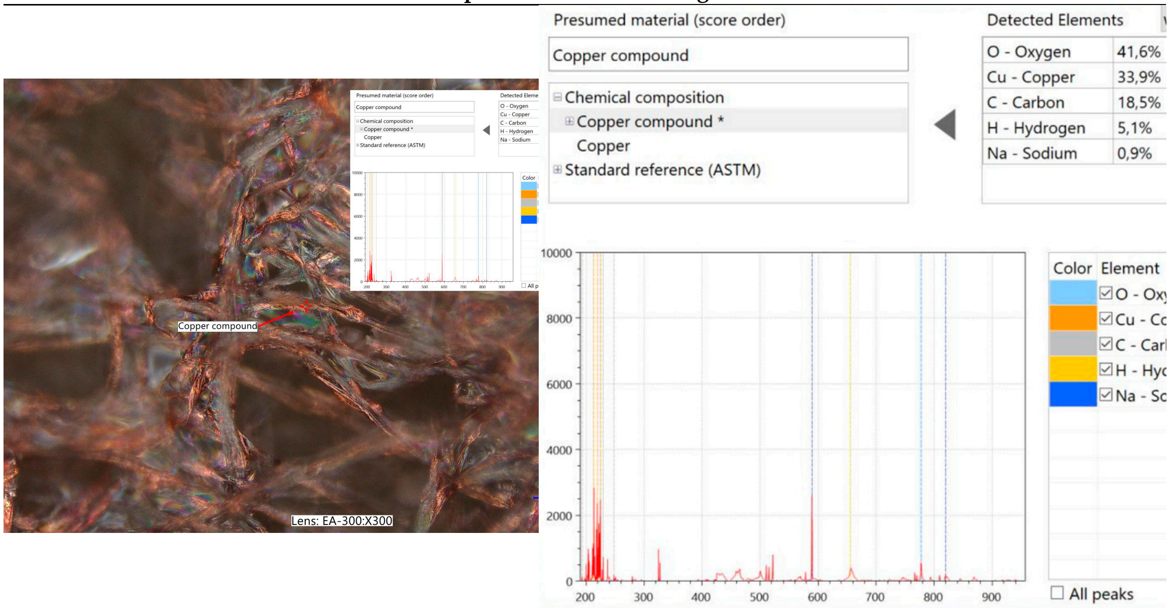

Figure S2.3. Elemental analysis of the **CNW-Cu^0(0.44)/Alg^-Na^+** sample using digital microscopy and Laser-Induced Breakdown Spectroscopy (LIBS).

Sample: CNW-Cu^0(0.44)/ALG^-Ca^{2+}(1.4)

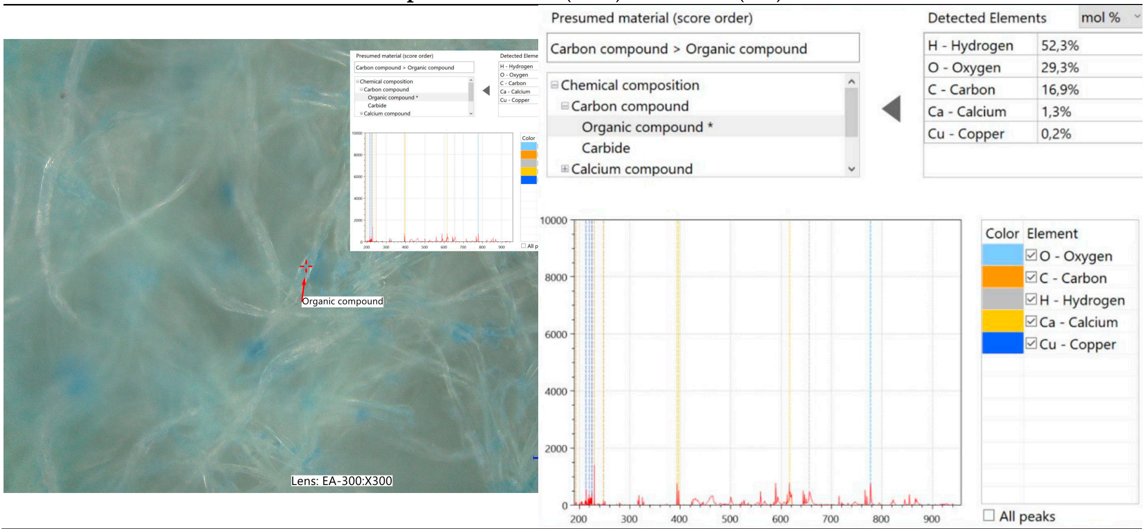

Figure S2.4. Elemental analysis of the CNW-Cu^0(0.44)/ALG^-Ca^{2+}(1.4) sample using digital microscopy and Laser-Induced Breakdown Spectroscopy (LIBS).

Sample: CNW-Cu/$ALG^{-}Ca^{2+}$(2.2)

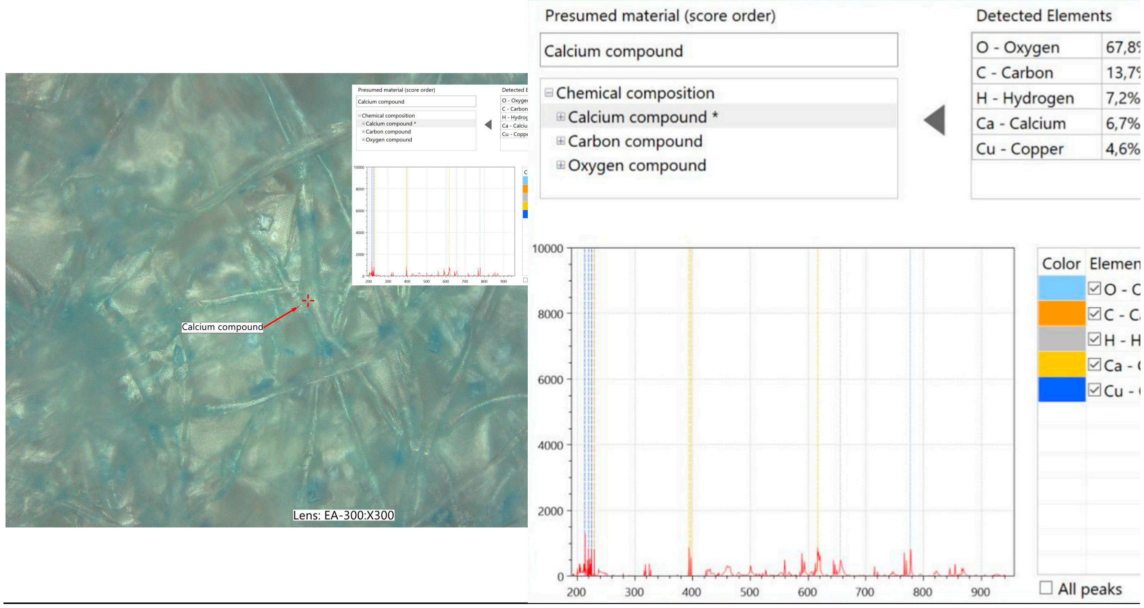

Figure S2.5. Elemental analysis of the CNW-Cu^{0}(0.44)/$ALG^{-}Ca^{2+}$(2.2) sample using digital microscopy and Laser-Induced Breakdown Spectroscopy (LIBS).

Figure S3. Scanning electron microscopy and elemental analysis (EDS).

**Sample:** CNW-Cu^0(0.4)/ALG^-Na^+		
**Magnification**		
500 x	5000 x	10000 x

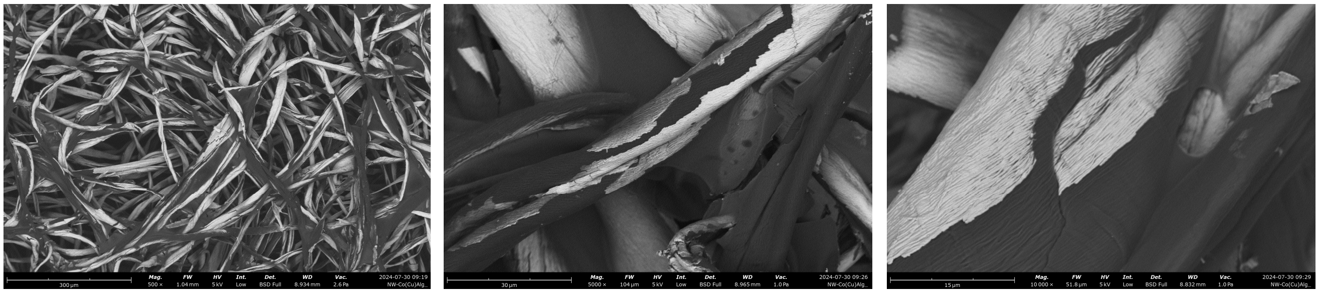

Figure S3.1. Scanning electron microscope (SEM) images of CNW-Cu^0(0.4)/ALG^-Na^+. Magnification: 500x; 5000×; 10000×.

**Sample:** CNW-Cu^0(0.4)/ALG^-Ca^{2+}(1.4)		
**Magnification**		
500 x	5000 x	10000 x

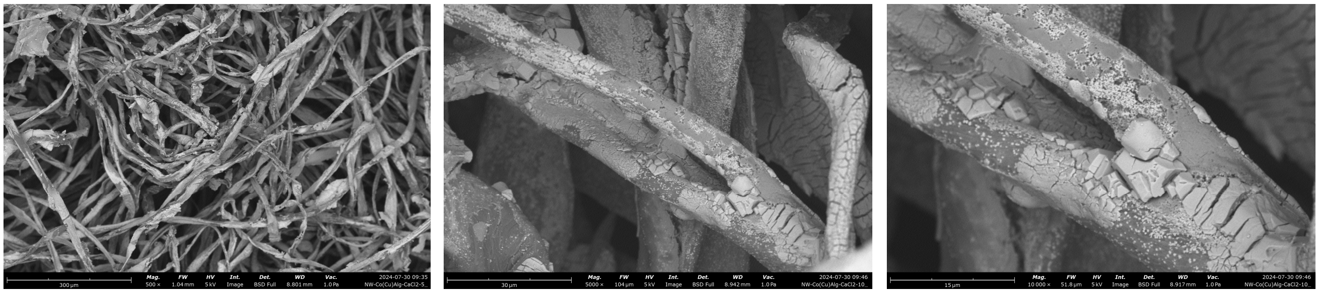

Figure S3.2. Scanning electron microscope (SEM) images of CNW-Cu^0(0.4)/ALG^-Ca^{2+}(1.4). Magnification: 500x; 5000×; 10000×.

**Sample:** CNW-Cu^0(0.4)/ALG^-Ca^{2+}(2.2)		
**Magnification**		
500 x	5000 x	10000 x

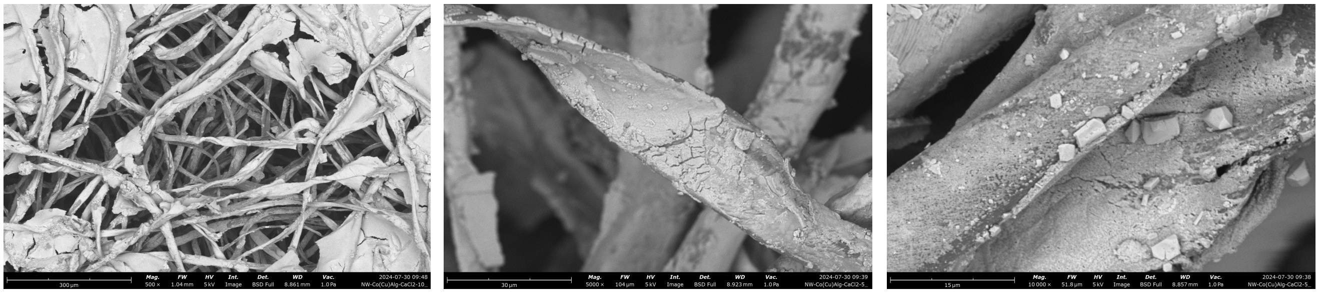

Figure S3.3. Scanning electron microscope (SEM) images of CNW-Cu^0(0.4)/ALG^-Ca^{2+}(2.2). Magnification: 500x; 5000×; 10000×.

Figures S4. Energy dispersive X-ray spectroscopy (EDS) experimental data for **CNW-Cu^0/Alg^-Na^+ , CNW-Cu^0/Alg^-Ca^{2+}(1.4)** and **CNW-Cu^0/Alg^-Ca^{2+}(2.2).**

Sample: CNW-Cu^0/Alg^-Na^+

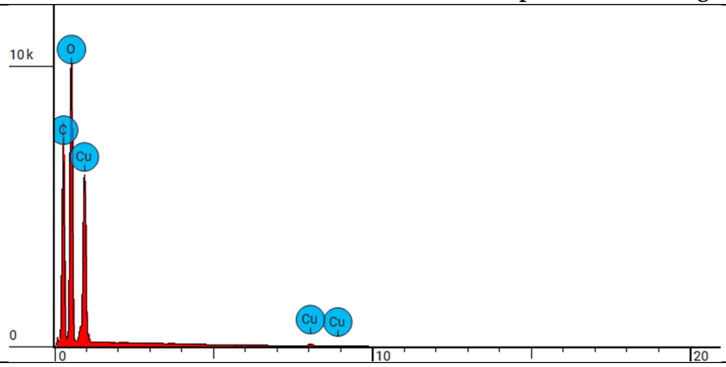

Element Name	Atomic Conc.	Weight Conc.
Carbon	42.070	26.200
Oxygen	47.489	39.400
Copper	10.441	34.400

Sample: CNW-Cu^0/Alg^-Ca^{2+}(1.4)

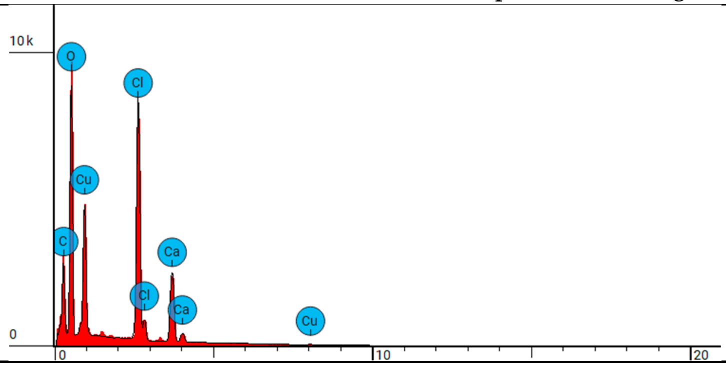

Element Name	Atomic Conc.	Weight Conc.
Carbon	23.145	11.635
Oxygen	40.438	27.081
Chlorine	21.220	31.494
Calcium	10.822	18.154
Copper	4.375	11.635

Sample: CNW-Cu^0/Alg^-Ca^{2+}(2.2)

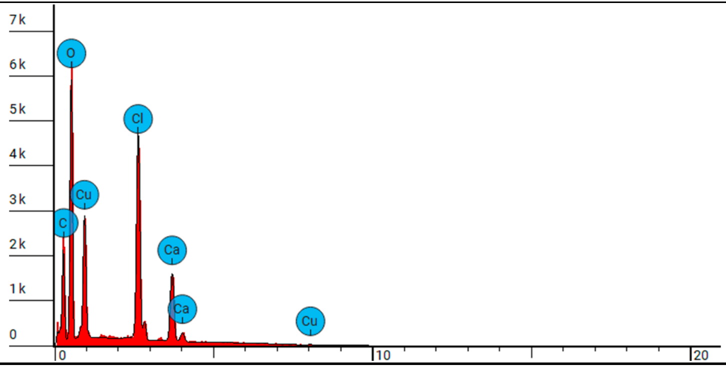

Element Name	Atomic Conc.	Weight Conc.
Carbon	26.491	13.886
Oxygen	40.914	28.571
Chlorine	18.074	27.972
Calcium	10.451	18.282
Copper	4.071	11.289

Figure S5. Obtained N_2 adsorption-desorption isotherms for: (a) CNW; (b) CNW-Cu^0(0.44); (c) CNW-Cu^0(0.44)/ALG-Na^+; (d) CNW-Cu^0(0.44)/ALG-Ca^{+2}(1.4); (e) CNW-Cu^0(0.44)/ALG-Ca^{+2}(2.2) samples.

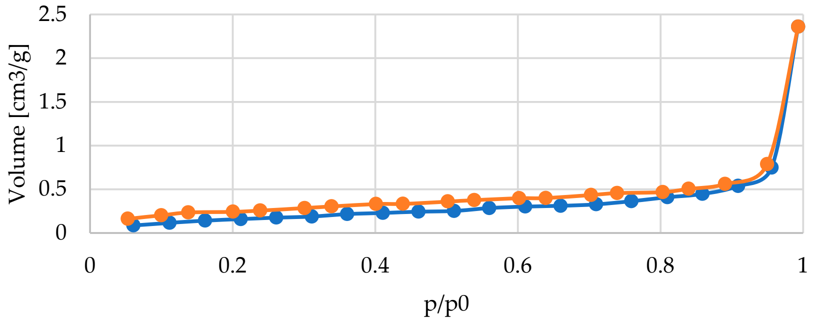

(a)

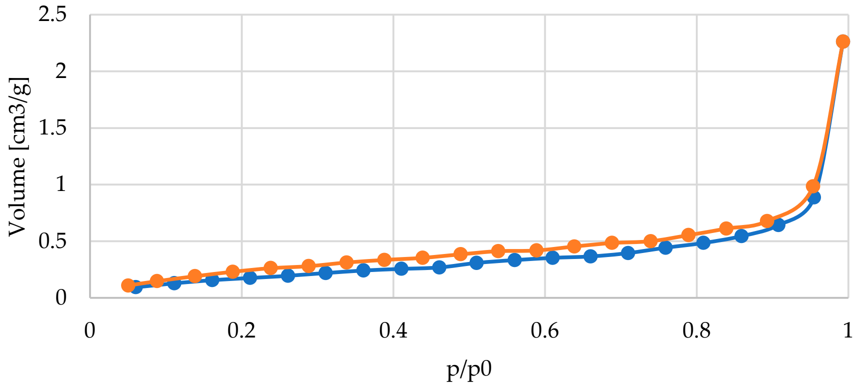

(b)

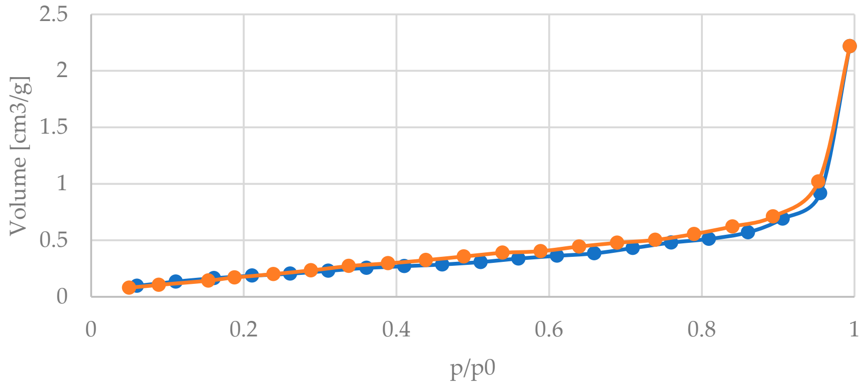

(c)

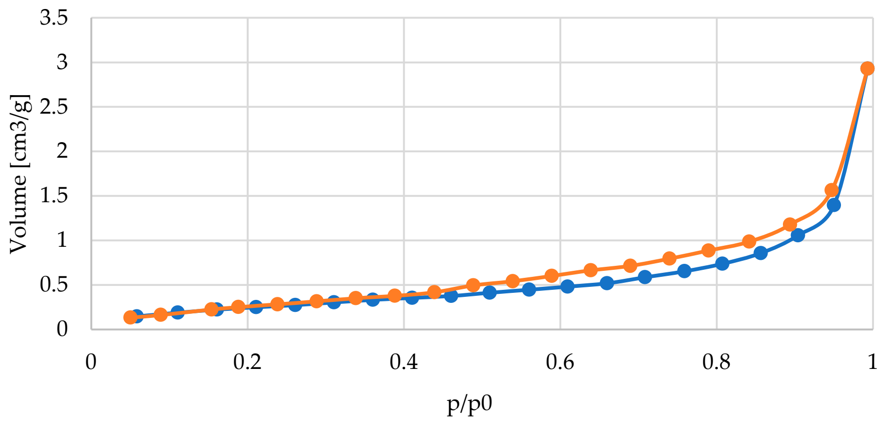
Volume [cm3/g]
p/p0
0
0.5
1
1.5
2
2.5
3
3.5
0.2
0.4
0.6
0.8

(d)

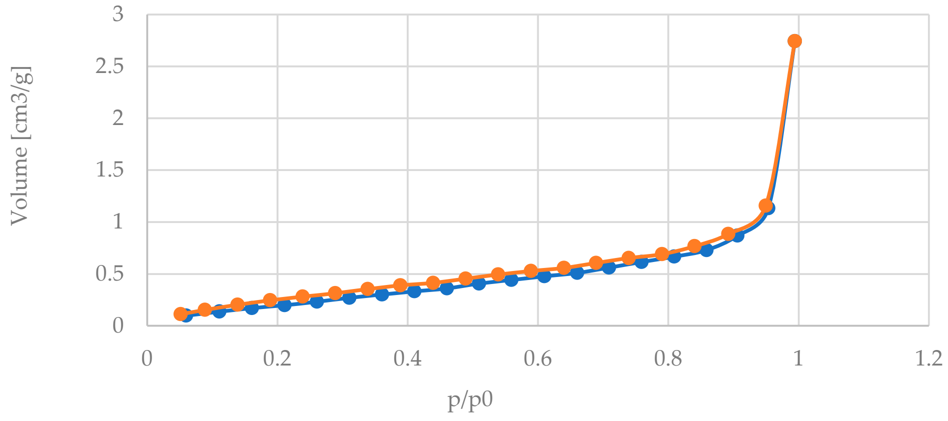
Volume [cm3/g]
p/p0
0
0.5
1
1.5
2
2.5
3
0.2
0.4
0.6
0.8
1.2

(e)
